# Mitigation Strategies against Antibody Aggregation Induced by Oleic Acid in Liquid Formulations

**DOI:** 10.1021/acs.molpharmaceut.4c00754

**Published:** 2024-10-24

**Authors:** Dominik Zürcher, Klaus Wuchner, Paolo Arosio

**Affiliations:** † Department of Chemistry and Applied Biosciences, Institute for Chemical and Bioengineering, ETH Zürich, 8093 Zürich, Switzerland; ‡ Cilag GmbH International, a Division of Johnson & Johnson TDS-Biologics, Analytical Development, 8200 Schaffhausen, Switzerland

**Keywords:** monoclonal antibody(s), oleic acid, surfactant degradation, polysorbates, free fatty acids, interfaces, protein formulation(s), protein particles and aggregation

## Abstract

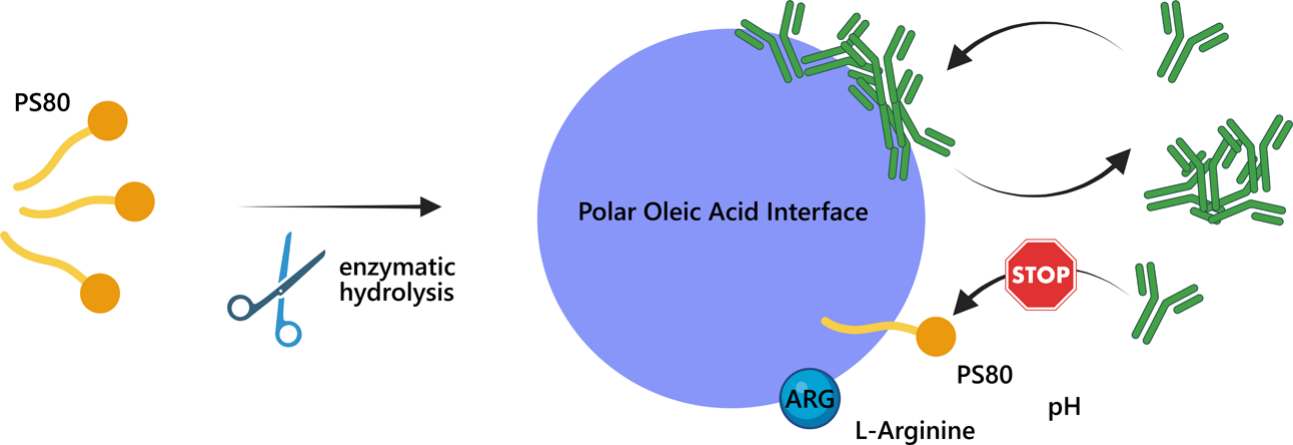

Polysorbates 20 and 80 (PS20 and PS80) are commonly used in the formulations of biologics to protect against interfacial stresses. However, these surfactants can degrade over time, releasing free fatty acids, which assemble into solid particles or liquid droplets. Here, we apply a droplet microfluidic platform to analyze the interactions between antibodies and oleic acid, the primary free fatty acid resulting from the hydrolysis of PS80. We show that antibodies adsorb within seconds to the polar oleic acid–water interface, forming a viscoelastic protein layer that leads to particle formation upon mechanical rupture. By testing two different monoclonal antibodies of pharmaceutical origin, we show that the propensity to form a rigid viscoelastic layer is protein-specific. We further demonstrate that intact PS80 is effective in preventing antibody adsorption at the oleic acid–water interface only at low antibody concentrations and low pH, where oleic acid is fully protonated. Importantly, introduction of the amino acid l-arginine prevents the formation of the interfacial layer and protein particles even at high antibody concentrations (180 mg mL^–1^). Overall, our findings indicate that oleic acid droplets in antibody formulations can lead to the formation of protein particles via an interface-mediated mechanism. Depending on the conditions, intact PS80 alone might not be sufficient to protect against antibody aggregation. Additional mitigation strategies include the optimization of protein physicochemical properties, pH, and the addition of arginine.

## 1 Introduction

The stability of therapeutic proteins against aggregation is a major quality concern during the drug lifecycle, as the presence of particles is highly restricted by regulatory authorities due to potential adverse health effects.^1−3^ An important pathway for protein particle formation proceeds through adsorption, conformational perturbation, film formation, and rupture at hydrophobic interfaces such as air and silicone oil.^4−10^ These interfaces are ubiquitous in the processing and delivery of biologics and often act in synergy with hydrodynamic flow.^11^

The nonionic surfactants polysorbate 20 and 80 (PS20 and PS80, respectively) are the most common stabilizers in parenteral formulations of biologics to prevent interfacial adsorption of the drug by competing for the interface.^12−14^ The main component of polysorbates is an ethoxylated sorbitan headgroup that is esterified with 1–4 fatty acids (FAs) of variable chain lengths. Oleic acid (OA) with a chain length of 18 is the most abundant FA in PS80, with a minimum content of 58.0% required by the European, Japanese, and United States Pharmacopoeias for multicompendial PS80, and actual levels ranging from 68 to 97%.^15,16^

A major limitation of PS20 and PS80 is their susceptibility to degradation by oxidation and hydrolysis. The latter degradation pathway is primarily catalyzed by trace amounts of host cell proteins (HCPs) such as esterases and lipases and results in the release of free fatty acids (FFAs).^17−19^ Given the typical concentration of PS80 in pharmaceutical formulations (∼0.5 mg mL^–1^)^20^ and the low solubility limit of oleic acid of 2–5 μg mL^–1^,^21^ the hydrolysis of even a small fraction (approximately 5–10%) of intact PS80 could lead to oleic acid concentrations that exceed its solubility limit.

Above their solubility limits, FFAs undergo phase separation, causing the formation of particles or droplets at ambient conditions, according to their melting points.^22^ Although it has been suggested that the presence of FFAs could destabilize proteins and promote the formation of proteinaceous particles, the mechanisms underlying this process remain unclear.^21,23−26^

This study focuses on OA, the primary FFA resulting from PS80 hydrolysis, which is found as liquid droplets above its melting temperature of 13–14 °C.^27^

While the p*K*_a_ value for most short-chain carboxylic acids in water is approximately 4.8 , significantly higher apparent p*K*_a_ values ranging from 5.7 to 9.8 were reported for OA.^22,27−30^ This effect is attributed to the self-association of fatty acid molecules, leading to complexes with high negative surface charge densities, which exhibit a complex, pH-dependent phase behavior.^28,31−34^ In the pH range of 4.8–8.0 reported for commercially available antibody formulations,^20^ OA polar head groups are therefore in equilibrium between the protonated and deprotonated forms. These considerations are relevant because the polarity of different oil–water interfaces has previously been shown to affect the interfacial adsorption behavior of globular proteins and surfactants.^35−37^ While the interaction between therapeutic proteins and fluid hydrophobic interfaces, such as silicone oil (SO)- and air–water interfaces, has been extensively investigated, there has been considerably less focus on interactions between therapeutic proteins and FFAs.

Recent approaches involved spiking formulations with OA or hydrolyzed PS80 before performing quiescent incubation or agitation studies in the presence of air–water interfaces.^21,25^ These methodologies, however, can pose challenges in identifying and characterizing destabilizing mechanisms due to the lag time between the application of stress and subsequent analysis.

In this work, we apply a recently developed microfluidic platform^38^ to investigate the interactions of therapeutic antibodies at concentrations up to 180 mg mL^–1^ with the liquid OA–water interface on short time scales. We further explore the effect of varying levels of intact PS80, sodium chloride, guanidinium hydrochloride, l-histidine (l-His), l-lysine (l-Lys), and l-arginine (l-Arg) at different pH values and consider two distinct mAbs of biopharmaceutical origin.

In analogy with recent findings obtained at the SO–water interface,^38^ we show that antibodies rapidly adsorb to the polar liquid OA interface, forming a viscoelastic protein layer that can lead to particle formation upon mechanical rupture. We find that the propensity to form this viscoelastic protein layer varies for different antibodies . We further demonstrate that the effectiveness of intact PS80 in preventing antibody adsorption is influenced by surfactant and antibody concentrations as well as formulation pH. Importantly, we find that the amino acid l-Arg effectively prevents interfacial layer and particle formation even at high antibody concentrations.

## 2 Materials and Methods

### 2.1 Materials

Recombinant humanized monoclonal antibodies mAb1 and mAb2 (Janssen, Schaffhausen, Switzerland) were provided in stock formulations containing >150 mg mL^–1^ protein. Unless stated otherwise, the samples were prepared in buffer containing 6% trehalose dihydrate (abcr GmbH, Karlsruhe, Germany), 44 mM sodium phosphate dibasic (Sigma-Aldrich, reag. Ph. Eur., St. Louis, MO, USA), and 10 mM citric acid (Sigma-Aldrich, reag. Ph. Eur., St. Louis, MO, USA) at pH 6.4 or 5.0. Alternatively, they were prepared in buffer containing 6% trehalose dihydrate and 25 or 130 mM l-His (Sigma-Aldrich, reag. Ph. Eur., St. Louis, MO, USA) at pH 6.4. Buffer exchanges were performed by diluting stock formulations in the target buffer and performing dialysis in either 500 μL membrane centrifugal concentrators (MWCO 50 kDa, Vivaspin 500, Sartorius, UK), dialysis cassettes (Slide-A-Lyzer, MWCO 7 kDa, Thermo Scientific, IL, USA), or using 10 mL Float-A-Lyzer devices (MWCO 100 kDa, Sigma-Aldrich, USA) according to the manufacturer’s instructions in 1 L of target buffer with at least one intermediate buffer change before measuring the pH values of dialyzed solutions.

The buffers were prepared using ultrapure water (Milli-Q Synergy Water Purification System, Merck Millipore, MA, USA) and filtered using a Nalgene vacuum filtration system (ThermoFisher Scientific, USA) and 0.45 μm Durapore PVDF filter membranes (Merck, Germany). Super refined PS80 was obtained from Croda Inc. (Edison, New Jersey, USA). PS80 stock solutions were prepared in buffer at pH 6.4 at 1% (w/v) and stored at −20 °C as 1 mL aliquots, thawed on the day of use, and further diluted to prepare the formulations. Surfactant concentrations are reported hereafter as % (w/v). Oleic acid (97%, Acros Organics, USA) was stored at 5 °C and thawed at room temperature on the day of use. mAb samples were filtered using 0.2 μm cutoff syringe filters (Millex Syringe Driven Filter Unit, Japan). The pH of buffers (6% trehalose dihydrate, 44 mM sodium phosphate dibasic, and 10 mM citric acid, pH 6.4) containing l-Arg, l-His, l-Lys, and guanidinium hydrochloride (GdnHCl) (BioUltra, Sigma-Aldrich, St. Louis, MO, USA) was adjusted using aqueous HCl. 8-Anilino-1-naphtalenesulfonic acid (ANS) was purchased from TCI Europe, Belgium. Protein samples were supplemented with ANS at 25 μM from a 500 μM aqueous stock solution, prepared from 10 mM ANS in DMSO (ACROS Organics, 99.7% extra dry over molecular sieves, Thermo Scientific, Waltham, MA, USA) on the day of the experiment, and used within a day.

### 2.2 Antibody Labeling

mAb1 and mAb2 were labeled at pH 6.4 with an Alexa Fluor 647 (AF647) *N*-hydroxy succinimidyl (NHS) ester (Conjugation Kit, Lightning-Link, abcam, Cambridge, UK) following the supplier’s specifications. Briefly, 100 μL of antibody at 1 mg mL^–1^ supplemented with 10 μL of modifier reagent was incubated with lyophilized dye for 1 h at ambient temperature in the absence of light before the addition of 10 μL of quencher reagent. The conjugates were stored at 5 °C and used without further purification. mAb formulations at 30 mg mL^–1^ were supplemented with labeled mAb1-Alexa Fluor 647 to achieve a molar ratio of 1:700 of the labeled and unlabeled antibodies.

### 2.3 Fabrication and Operation of Microfluidic Chips

Master molds were produced in-house by spin-coating SU-8 (MicroChemicals, Ulm, Germany) onto a silicon waver, followed by soft baking. Subsequently, the silicon waver was exposed to ultraviolet light with a mask incorporating the chip layout (designed in Auto-CAD 2021) to induce local polymerization prior to postbaking. Standard soft lithography was performed to replicate the chip geometry by pouring a 10:1 mixture of polydimethylsiloxane (PDMS) and curing agent (Sylgard 184, Dow Corning, Midland, MI) onto the mold, followed by degassing (1 h) and baking (2 h at 65 °C). The microfluidic chip was bonded to glass slides (Menzel, Braunschweig, Germany) after plasma activation (ZEPTO plasma cleaner, Diener Electronics, Ebhausen, Germany). The microfluidic chips were used within 2 h after bonding. The chip had two inlets, one outlet, and a flow focusing nozzle. The height of the channel was 50 μm, and the width of the nozzle and the first section of the channel was 100 μm. The second section comprised linear channels with a width of 70 μm and 58 expansion regions measuring 200 μm in width and length. The fluid flow inside the channels was modulated using an external syringe pump (Cetoni neMESYS, Cetoni GmbH, Korbussen, Germany) that controlled the movement of a plunger of a 500 μL unsiliconized glass syringe (Hamilton, Reno, NV, USA). The connection to the corresponding inlet of the microfluidic chip was achieved via PTFE tubing (Adtech Polymer Engineering Ltd., Stroud, UK). The protein formulation and the oleic acid flow rates were maintained at 1.2 and 0.125 μL min^–1^, respectively. These flow rates correspond to a droplet residence time of 35 s within the microfluidic chip. Image acquisition was started after stable drop formation was established for 15 min, and droplets were collected using a 200 μL gel-loading pipet tip inserted into the chip outlet. Microfluidic chips were designed for single use, and the operation required a minimum sample volume of 50 μL.

### 2.4 On-Chip Quantification of Droplet Deformation

The shape and dimensions of the droplets were extracted from microscopy images captured simultaneously at different expansion regions at a 4× magnification during the chip operation. A minimum of 200 images were acquired per experimental condition and expansion region. The characterization of droplet shapes within the expansion regions involved postimage binarization using a custom MATLAB algorithm, which yielded the width (*w*) and height (*h*) of the droplets. The dimensionless droplet deformation parameter *D* was then computed as follows:
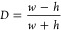
1*D* is equal to 0 for perfect circles. The mean and standard deviation of *D* were determined for a minimum of five droplets per region and condition. Additional details are provided in the Supporting Information (Figure S1).

### 2.5 Acquisition of Microscopy Images

A Ti2–U inverted microscope (Nikon, Switzerland) equipped with an LED light source (Omicron Laserage Laserprodukte GmbH, Germany), a camera (Zyla sCMOS 4.2P-CL10, Andor, UK), and Nikon CFI Plan Fluor Objectives (4×, 10×, and 20× magnification) was used to acquire images. The acquisition was started after 15 min of stable droplet formation.

### 2.6 Off-Chip Characterization of Samples

Brightfield microscopy images of samples were captured by pipetting approximately 10 μL of the sample, which was collected at the chip outlet using gel-loading tips, onto a glass slide, followed by image acquisition. The extrinsic dye ANS was detected by sample excitation with 365 nm and selecting the emission between 417 and 477 nm. ANS fluorescence images were acquired at 20× magnification, 200 ms integration time, and 770 mW excitation laser power. Alexa Fluor 647 fluorescence images were acquired at 20× magnification, 200 ms (off-chip) or 2 ms (on-chip) integration time, and 260 mW (off-chip) or 425 mW (on-chip) excitation laser power (excitation wavelength: 617 nm, integrated emission wavelengths: 640–690 nm).

### 2.7 Measurement of Diffusion Interaction Parameter k_D_

The stock solutions of mAb1 and mAb2 at pH 6.4 were diluted to a range of concentrations (4, 7, 10, 13, 17, and 20 mg mL^–1^), filtered (0.02 μm, Whatman Anotop, sterile, inorganic membrane filter, Cytiva, Germany), and triplicate measurements of mutual diffusion coefficients *D*_m_ were performed by dynamic light scattering (DLS) at 450 nm and 25 °C using a Prometheus Panta (Nanotemper Technologies, Munich, Germany). Samples were prepared by filling standard capillaries with 10 μL of the solution. The relationship of the diffusion interaction parameter *k*_D_ with the mutual diffusion coefficients *D*_m_ measured at different antibody concentrations *c* and the self-diffusion coefficient *D*_0_ is given by the following equation.^39^
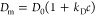
2

The values for *k*_D_ were determined by linear regression of the data of *D*_m_ vs *c* to eq 2, and their standard deviations were calculated by propagating the standard errors of the fitted coefficients. Data and linear fit are shown in Figure S2.

### 2.8 Protein Thermal Stability Characterization

The thermal stability of mAb1 and mAb2 at pH 6.4 was determined using a Prometheus Panta (Nanotemper Technologies, Munich, Germany) and standard capillaries filled with 10 μL of an antibody solution at 7 mg mL^–1^. The intrinsic fluorescence at 330 and 350 nm upon excitation at 280 nm was measured while the sample was heated from 20 to 90 °C at a rate of 1 °C min^–1^. In parallel, the hydrodynamic radius of the sample was monitored using DLS. Data were analyzed using Nanotemper’s analysis software by applying a 2-state fit to the fluorescence ratio at 350 and 330 nm. The unfolding curves of the two mAbs are shown in Figures S3 and S4.

## 3 Results and Discussion

### 3.1 Polarity and pH-Dependent Phase Behavior of Oleic Acid

**Figure 1:**
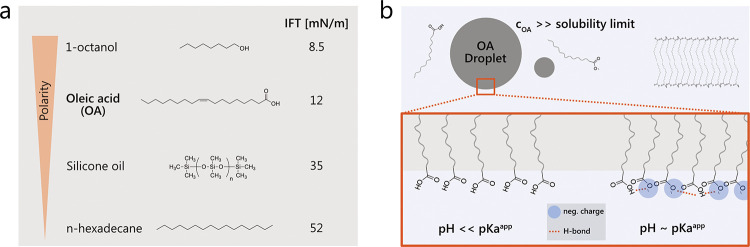
Polarity and phase behavior of oleic acid (OA). (a) Polarity ranking of OA and other hydrophobic oils according to the interfacial liquid tension (IFT). Values for IFT of 1-octanol, OA, silicone oil, and *n*-hexadecane were taken from the literature.^37,40,41^ (b) Phase behavior of OA above its solubility limit and melting temperature in the relevant pH range of antibody formulations (4.8–8).^20^ Partial ionization of the carboxylic head groups can result in an increase of the interfacial net-charge and intermolecular ion–dipole interactions.^27^

Figure 1a shows the molecular structures of OA and other hydrophobic liquids with a polarity ranking according to the interfacial liquid tension (IFT).^42^ This interfacial property depends on molecular characteristics including dipole moment and ionization potential and has previously been shown to predict the adsorption behavior of globular proteins and surfactants, as well as the strength of adsorbed viscoelastic protein layers at liquid–liquid interfaces.^35−37^Figure 1b schematically illustrates the phase behavior of OA above its solubility limit (reported values range between 2 and 5 μg mL^–1^ in pharmaceutical buffers)^43^ in the pH range characteristic for antibody formulations (4.8–8.0)^20^ and above its melting temperature of 13–14 °C.^27^ Prolonged exposure of mAb products to temperatures above the OA melting point can occur in scenarios such as temperature stability and photostability studies or during analytical testing and handling of drug products such as administration. Oil droplets and lamellar assemblies were observed in aqueous environments at pH < 8.^28,44^

The apparent p*K*_a_ value of palmitic acid (with a chain length of 16), an FFA related to the degradation of PS20, was determined to be 7 in formulations containing both antibodies and polysorbates.^22^ This finding provides a lower bound of ≥7 for the apparent p*K*_a_ value of OA under pharmaceutically relevant solution conditions, considering that the value tends to increase with the length of the carbon chain. At pH values ≤5, significantly lower than the apparent p*K*_a_ (∼7), OA can thus be expected to be fully protonated, whereas at higher pH values, OA is expected to be partially or fully ionized (Figure S5). Deprotonation causes an increase in the interfacial net-charge and attractive ion–dipole interactions between OA carboxyl groups, which results in a decrease in the intermolecular distances between OA molecules at the interface and potentially modulates the interaction strength between the OA interface and adsorbing protein and surfactant molecules.^27^

### 3.2 Antibodies Rapidly Adsorb and Form a Viscoelastic Layer on Oleic Acid Droplets

We have recently developed a microfluidic droplet device capable of simultaneously probing the adsorption, viscoelastic layer formation, and aggregation of proteins at the SO–water interface on short time scales.^38^

**Figure 2:**
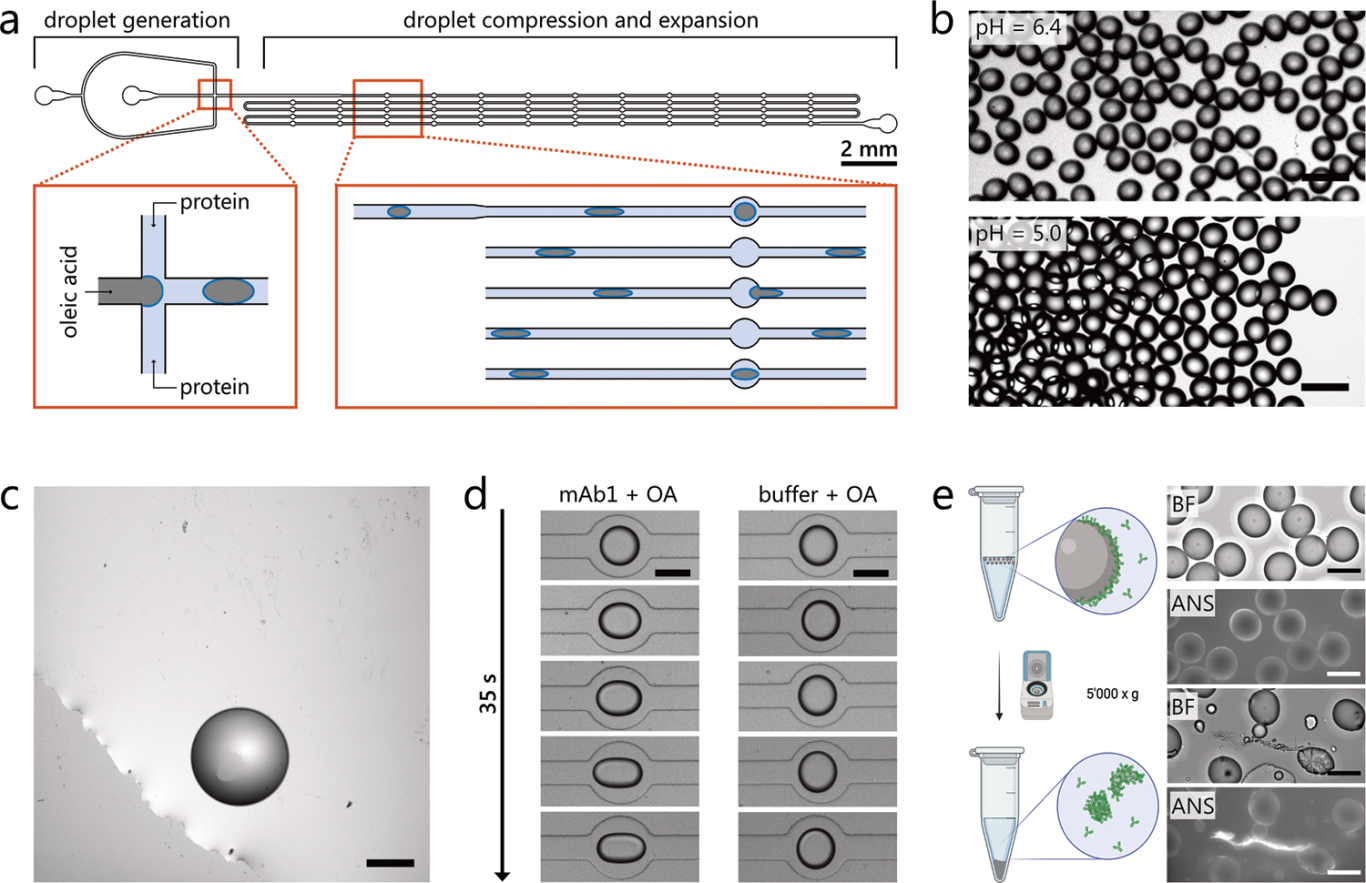
Schematic illustration of the microfluidic chip and microscopy images of samples obtained on- and off-chip. (a) Oleic acid (OA) droplets in protein formulations are formed in the 100 μm flow focusing nozzle . The channel narrows from 100 to 70 μm to compress the droplets before entering the expansion regions (with a width of 200 μm). (b) OA droplets formed in the presence of 30 mg mL^–1^ mAb1 in the same buffer at pH 6.4 and 5.0 are colloidally stable, exhibiting rather monodisperse diameters of about 100 μm. The scale bar is 200 μm. (c) Off-chip analysis of OA droplets formed in buffer at pH 6.4 without protein, showing an OA droplet with the size of approximately 500 μm due to coalescence of smaller droplets. The scale bar is 200 μm. (d) In the presence of mAb1 (30 mg mL^–1^, pH 6.4), OA droplets show increasing elongational deformation as they travel through the chip due to the formation of a viscoelastic protein layer that restricts their relaxation into a circular shape. In the presence of buffer alone at pH 6.4, droplets can fully relax. The scale bar is 100 μm. (e) Mechanical perturbation of the viscoelastic protein layer around OA droplets formed in the presence of 30 mg mL^–1^ mAb1 at pH 6.4 via centrifugation (5 min, 5000 × *g*) leads to buckling of the protein layer and causes the generation of ANS positive, proteinaceous particles. The scale bar is 100 μm.

A schematic representation of the microfluidic chip is shown in Figure 2a. Monodisperse micrometer-sized OA droplets are formed in protein formulations at pH 6.4 and 5. OA droplets travel as vertically squeezed plugs inside the channel, wherein they experience a total of 58 expansion and compression cycles. These regions allow for the repeated shape relaxation of the protein-loaded droplet, while at the same time enabling the detection of the rheological response of the droplet interface to the flow field. Brightfield microscopy images are acquired at multiple positions corresponding to droplet residence times in the range of 0.3 and 35 s. The samples can be further analyzed off-chip by collecting droplets at the outlet of the microfluidic chip.

We first generated OA droplets in the presence of 30 mg mL^–1^ mAb1 at both pH 5 and 6.4, which remained colloidally stable when collected at the chip outlet (Figure 2b). In contrast, droplets generated in buffer readily merged into larger ones (Figure 2c).

These observations demonstrate the rapid adsorption of antibodies at the interface of OA droplets, which effectively stabilizes them against coalescence. The protein adsorption results in the progressive formation of a viscoelastic protein layer, which is demonstrated by the restricted droplet relaxation into discs within expansion regions (Figure 2d and Movie S1). This elongational deformation was absent when OA droplets were formed in the buffer alone. The transition from fluid-like to viscoelastic behavior is likely driven by conformational rearrangements and subsequent interactions between adsorbed proteins.^45^ This phenomenon has previously been shown to occur at air–water and various oil–water interfaces, resulting in rigid protein layers that modulated the deformation of oil droplets subjected to shear flows.^38,46−50^ The formation and presence of viscoelastic antibody layers at fluid interfaces are critical in the context of biopharmaceuticals, as they precede the formation of proteinaceous particles upon mechanical rupture.^7,9,10^ Indeed, upon mechanical perturbation by centrifugation (5000 × *g*, 5 min) of OA droplets formed in the presence of mAb1, we observed the presence of wrinkles that formed via out-of-plane deformations (buckling) of the OA interface (Figure 2e).^51^ Moreover, particles were released that could be stained by the extrinsic dye ANS, which reports on protein unfolding and aggregation.^52^ Increasing the extent of mechanical perturbation by centrifugation led to a drastic increase in the formation of particles (Figure S6).

### 3.3 Effect of Oil Polarity and Protein Physicochemical Properties on Viscoelastic Layer Formation

The adsorption and formation of viscoelastic layers around liquid interfaces are influenced by the physicochemical properties of the protein. For instance, the globular protein beta-lactoglobulin (BLG) has been shown to form stronger viscoelastic layers than bovine serum albumin (BSA) at oil interfaces of different polarity.^46,53^ These differences have been attributed to the lower thermodynamic stability, smaller size, and more negative net charge of BLG compared to BSA. Differences have also been observed within the same class of therapeutic proteins (IgG1), showing stronger interfacial layers for the antibody with a higher propensity to form aggregates.^6^

We therefore compared the formation of viscoelastic layers of two mAbs of pharmaceutical origin, mAb1 and mAb2 , around oils of different polarities, namely OA and SO, the latter representing the most common liquid–liquid interface found in the context of biotherapeutic drug delivery.^5,6,10^ The interfacial stability of both mAbs has previously been evaluated and showed superior stability of mAb2 over mAb1 at all interfaces tested, including air–water and solid–water interfaces with varying levels of negative charge and hydrophobicity.^54^

The two IgG1s exhibited significant differences in zeta potentials and dipole moments and were similar in terms of computed hydrophobic patch areas, measured melting temperatures *T*_m_, and diffusion interaction parameters *k*_D_, which is considered a good predictor of problematic solution behavior such as viscosity and opalescence.^55^ The calculated and measured physicochemical properties of the two mAbs are summarized in Table 1.

**Table 1:** Selected Physicochemical Properties of mAb1 and mAb2^a^

protein	pI	zeta pot. [mV]	hydrophobic patch area	dipole moment	*k*_D_ [mL g^–1^]	*T*_m_ [°C]
mAb1 (IgG1)	9.2	+26.5	2425	2700	–5.55 ± 0.01	72.40 ± 0.03
mAb2 (IgG1)	7.6	+11.5	2325	900	–5.36 ± 0.01	70.00 ± 0.02

a Isoelectric point (pI), zeta potential, hydrophobic patch areas, and dipole moment were computed from their amino acid sequences and 3D structures. The diffusion interaction parameter *k*_D_ and melting temperatures *T*_m_ were experimentally measured in the same buffer at pH 6.4.

The isoelectric points of mAb1 and mAb2 were close to the upper and lower bounds of typical values (6.5–9.5) of approved mAbs with favorable solution behavior,^55^ whereas the negative *k*_D_ values indicated a tendency for self-association at higher concentrations. Both mAbs exhibited apparent melting temperatures higher than typical cutoff values used to flag problematic mAbs during developability assessment.^56^

**Figure 3:**
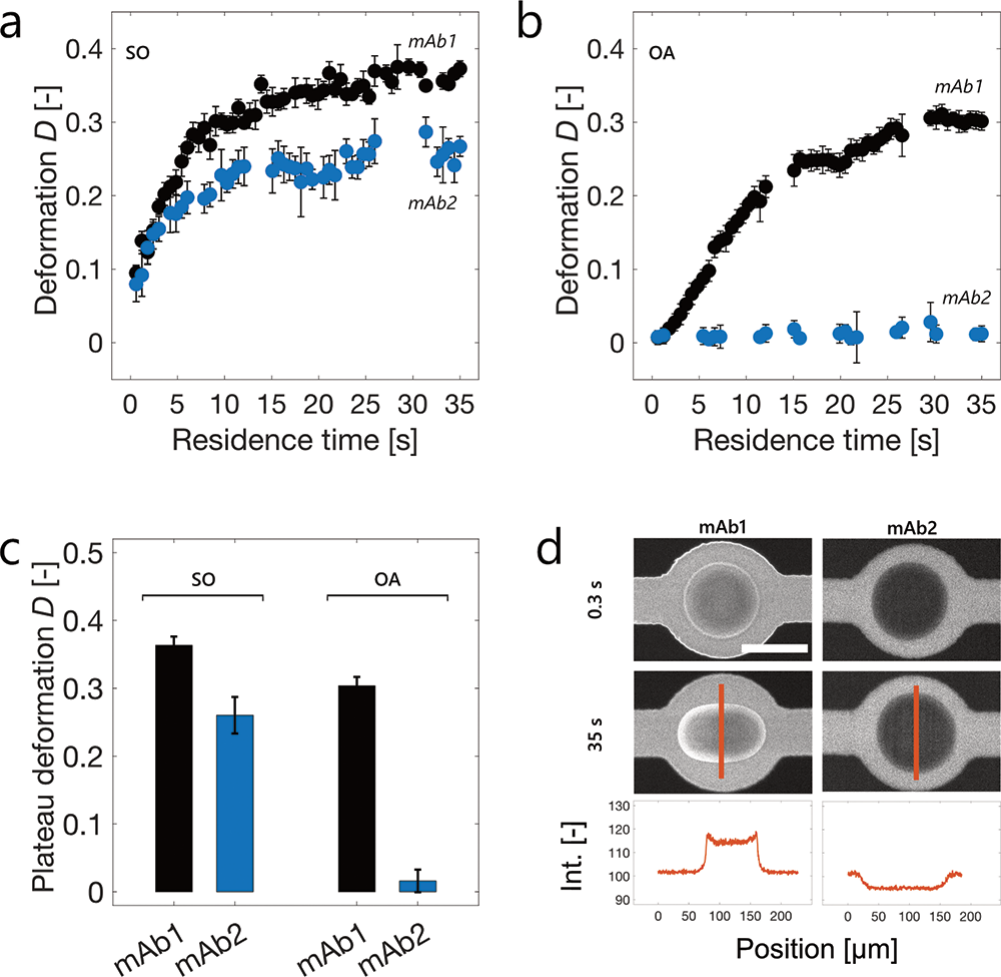
Formation of a viscoelastic protein layer around SO (a) and OA (b) droplets in a 30 mg mL^–1^ mAb1 or mAb2 formulation at pH 6.4. (c) Plateau values of the droplet deformation at the end of the chip, reporting on the strength of the viscoelastic protein layer. The deformation data of mAb1 at SO shown in (a) and (c) were taken with permission from ref (38). (d) Fluorescence microscopy images of OA droplets in the presence of mAb1 and mAb2 formulations, spiked with labeled mAb1-Alexa Fluor 647 and mAb2-Alexa Fluor 647, respectively, at the initial (0.3 s) and final (35 s) positions of the microfluidic chip. The fluorescence intensity of a representative droplet was quantified along its minor axis (orange line). The scale bar is 100 μm.

We quantified the extent of deformation of OA droplets formed in protein formulations in different expansion regions corresponding to residence times ranging between 0.3 and 35 s using the dimensionless parameter *D* = (*w* – *h*)/(*w* + *h*), where *w* and *h* represent the major and minor axes of the droplet, respectively (see Methods and Figure S1). Figure 3a–c shows the extent of deformation of SO and OA droplets in the presence of mAb1 and mAb2 at 30 mg mL^–1^ and pH 6.4. The data of mAb1 at the SO interface were taken from ref (38). The deformation of SO droplets increased with the residence time for both mAbs and reached a plateau after approximately 20–30 s, which can therefore be considered the characteristic time scale for viscoelastic layer formation under these conditions. At the SO interface, mAb2 showed significantly less deformation (−30%) compared to mAb1. Essentially, no layer formation was detected for mAb2 at the OA–water interface, while mAb1 formed a rigid layer whose strength showed a plateau after a similar time scale as with SO (Figures 3b,c).

We confirmed these results by spiking the samples with Alexa Fluor 647-labeled antibodies at a 1:700 ratio of labeled to unlabeled mAb and observing their on-chip adsorption at the OA–water interface. In agreement with the deformation data, OA droplets in the presence of mAb1 showed a bright fluorescence rim, whereas no adsorption of mAb2 could be detected (Figure 3d). Moreover, droplets formed in the presence of mAb2 underwent rapid coalescence events off-chip, further demonstrating negligible or no adsorption (Movie S2).

Next, we assessed whether the deformation data could correlate with particle formation under conditions close to those of product formulations. To this aim, we applied mechanical perturbation using a stress testing which simulates conditions close to drug administration by syringes.^11^ Specifically, OA droplets formed in mAb1 and mAb2 formulations were subjected to pumping cycles using pharmaceutical plastic syringes. Consistent with the droplet deformation data and the fluorescence signal measured on-chip, OA droplets formed in the mAb1 formulation shed proteinaceous particles into solution upon syringe pumping, while no particles were observed in the mAb2 formulation (Figure S7).

These data further agree with the previously reported superior stability of mAb2 at air–water and different solid–liquid interfaces.^54^ At pH 6.4, mAb2 has a lower net charge compared to that of mAb1, which, in combination with its significantly lower dipole moment, may reduce adsorption at the polar and negatively charged OA interface. Here, stability differences within antibodies may be more pronounced than at hydrophobic interfaces, such as air and SO–water, since proteins can populate more native conformations compared to non-native structures generated at hydrophobic interfaces. Protein surface properties are therefore important to modulate interactions at polar interfaces, including electrostatic and dipole–dipole interactions, but may have smaller effects on non-native, hydrophobic interactions at nonpolar interfaces.

The results illustrate that the destabilizing effect of OA is dependent on the specific mAb, and that care should be taken with antibodies that exhibit high zeta potentials and dipole moments in formulations at pH values where OA is expected to bear a net negative charge. Interestingly, the interfacial stability of the two mAbs differs despite their similar bulk stability behavior assessed by *k*_D_ and *T*_m_.

### 3.4 The Formation of a Viscoelastic Layer around OA Droplets is Modulated by pH and PS80

**Figure 4:**
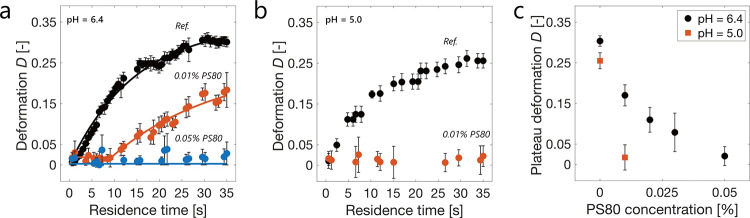
Effect of surfactants on the formation of a viscoelastic protein layer around the OA–water interface at different pH values. (a) Droplet deformation in the presence of 30 mg mL^–1^ mAb1 at pH 6.4 in the absence (“Ref.”) and presence of PS80 at 0.01% (6-fold cmc) and 0.05% (30-fold cmc). The solid lines represent a global fit based on a Langmuir adsorption model (*R*^2^ = 0.97, eqs S4–S6). (b) Same experiment as in (a) but at pH 5.0. (c) Plateau values of the deformation of OA droplets formed in 30 mg mL^–1^ mAb1 at pH 6.4 and 5.0 in the presence of PS80 in the range from 0.01 to 0.05%. The deformations over time at pH 6.4 for 0.02 and 0.03% PS80 concentrations are shown in Figure S8.

We then investigated the influence of pH and intact PS80 concentration on the kinetics and extent of the protein layer formation. The deformation of droplets increased with residence time and reached an apparent plateau after approximately 30 s (Figure 4a,b) at both pH 5.0 and 6.4. The deformation plateau value of mAb1 at pH 6.4 was higher than at pH 5, indicating that adsorbed proteins formed a slightly stronger viscoelastic layer when formed at the partially ionized OA interface, which may be caused by a denser packing of proteins.^57,58^ At pH 5, OA is expected to be fully protonated in contrast to pH 6.4, where a degree of dissociation of about 20% was computed using the Henderson–Hasselbalch relationship (Figure S5).

For the same mAb and buffer system, PS80 prevented the formation of a viscoelastic layer at the nonpolar SO interface at concentrations above its critical micelle concentration (cmc).^38^ In the presence of 0.01% PS80, a concentration 6-fold higher than its cmc (0.0017%^59^), the formation of a protein layer at the OA interface was only partially prevented at pH 6.4 (Figure 4a), whereas complete protection was observed at pH 5.0 (Figure 4b). A PS80 concentration of 0.05% (30-fold higher than cmc) was required to completely prevent the formation of a viscoelastic protein layer at pH 6.4. We note that in commercial antibody products, PS80 is formulated at concentrations ranging from 0.6- to 120-fold its cmc, with the majority equal or below 30-fold.^20^

The higher concentration of PS80 required to compete with antibody adsorption at the OA–water interface compared to the SO–water interface can be explained by the higher polarity, which leads to weaker interactions.^35^ Additionally, oils with increased polarity can interact more strongly with water via H-bonds and polar−π interactions.^60^ These attractive interactions result in a competition between the oil and surfactant molecule for interfacial adsorption, leading to a reduction in the maximum interfacial concentrations of the surfactant.^35^ At pH 6.4, partially ionized OA bears a net negative charge and exhibits increased polarity compared to pH 5, causing strong ion–dipole interactions between the ionized and protonated carboxyl groups on the OA interface with each other (Figure 1b) and surrounding water molecules.^27,28^ Thus, the affinity of the intact PS80 surfactant for the polar OA interface may be reduced, allowing antibody molecules to compete more effectively for interfacial adsorption. Consequently, mAbs form a viscoelastic layer, although with lower strength compared to a surfactant-free system, likely due to the presence of coadsorbed, intercalating PS80.^6,61^

We also tested intermediate PS80 concentrations between 0.01 and 0.05% at pH 6.4, observing a monotonic decrease of the plateau value with increasing PS80 concentrations (Figure 4c). Interestingly, for 0.01, 0.02, and 0.03% PS80 concentrations, the droplet deformation profiles exhibited an apparent lag phase during the first 10 s (Figures 4a and S8). This lag phase can be explained by considering the faster adsorption of PS80 compared to the mAb. The mAb is initially excluded from the interface as a result of the kinetic competition with the surfactant. Assuming that antibody adsorption is essentially irreversible and that surfactant adsorption is reversible and characterized by a high desorption rate constant, the antibody can subsequently accumulate at the interface and form a viscoelastic layer. This mechanism is supported by a model based on Langmuir adsorption which describes the experimental data (Figures 4a, S9–10, eqs S4–S6, and Table S1).

### 3.5 l-Arg Prevents Antibody Adsorption and Viscoelastic Layer Formation

In the previous section, we showed that, under certain buffer conditions and surfactant concentrations, intact PS80 alone may not be sufficient to protect antibodies at the OA–water interface.

**Figure 5:**
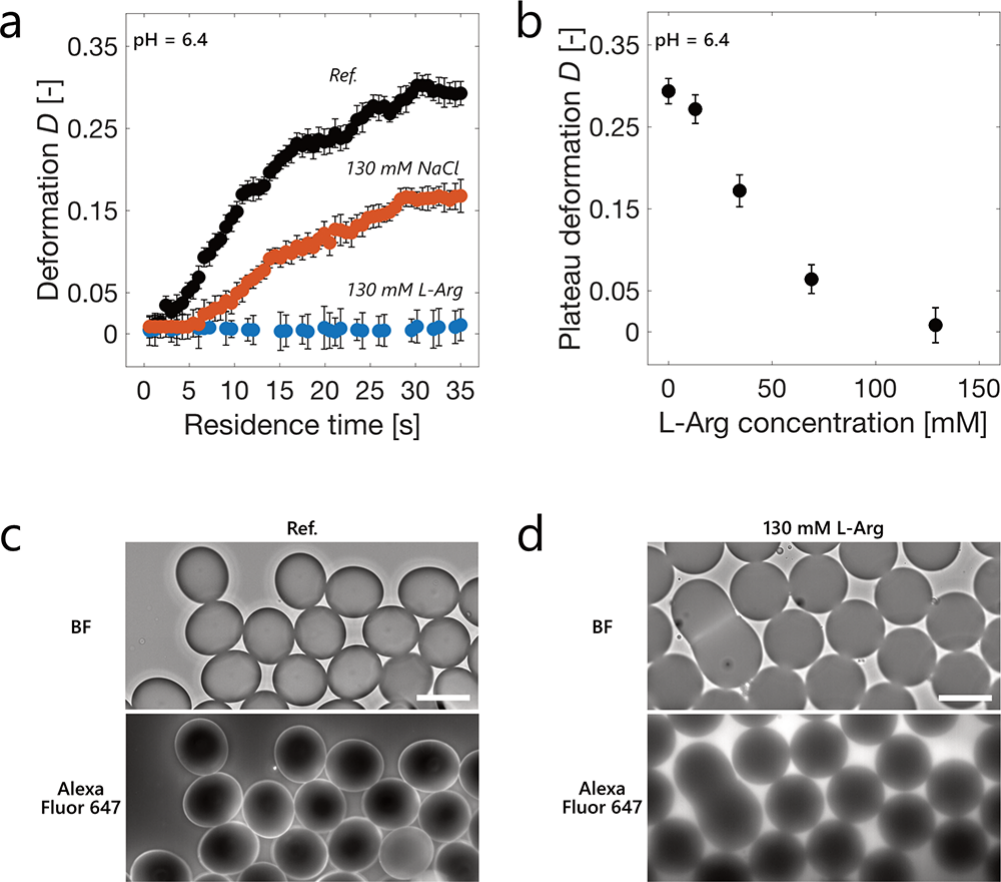
Effect of NaCl and l-Arg on the formation of a viscoelastic protein layer around the OA–water interface. (a) Deformation of OA droplets in 30 mg mL^–1^ mAb1 formulation at pH 6.4 in the absence (“Ref.”) and presence of 130 mM l-Arg or NaCl. (b) Plateau values of the deformation of OA droplets formed in 30 mg mL^–1^ mAb1 solution at pH 6.4 in the absence and presence of 13, 34, 70, and 130 mM l-Arg (full deformation data sets in Figure S12). (c, d) Brightfield (BF) and fluorescence microscopy images of OA droplets collected at the outlet of the microfluidic chip formed in mAb1 formulation spiked with labeled mAb1-Alexa Fluor 647 in the absence (c) and presence (d) of 130 mM l-Arg. Scale bars are 100 μm.

We therefore analyzed the effect of other excipients commonly present in the antibody formulations. The addition of 130 mM sodium chloride (NaCl) to a surfactant-free formulation decreased the strength of the viscoelastic layer but could not suppress its formation (Figure 5a). Even a 2-fold increase in the concentration (260 mM NaCl) led only to a further marginal decrease in the deformation plateau (Figure S11a). These results suggest that shielding of attractive electrostatic interactions between the interface and mAb1 alone may not be sufficient to prevent protein adsorption and layer formation.

We next investigated the effect of l-arginine (l-Arg), which is widely used in therapeutic antibody formulations due to its favorable effects on protein bulk properties, such as reduction of solution viscosity and aggregation.^62−65^ However, to the best of our knowledge, the effect of l-Arg on the antibody interfacial stability at high concentrations has not been investigated. l-Arg at pH 6.4 exhibits various features, including a positive net charge that can modulate electrostatic interactions, as well as a deprotonated carboxyl group, a protonated amino group, and a guanidinium group, which allow the formation of intermolecular H-bonds and interactions with aromatic side chains.^66^

We tested the effect of l-Arg at concentrations between 13 and 130 mM, which correspond to the typical range in approved antibody products.^64^ The time-dependent deformation values in the presence of 130 mM l-Arg at pH 6.4 in the absence of PS80 are shown in Figure 5a, while the plateau values at the end of the device as a function of l-Arg concentration are shown in Figure 5b (see Figure S12 for full data sets). Increasing the concentration of l-Arg led to a gradual decrease of the strength of the interfacial layer, whose formation was completely inhibited at 130 mM. Interestingly, the formation of the layer was not completely inhibited when the formulation was supplemented with either 130 mM guanidine hydrochloride (GdnHCl), l-lysine (l-Lys) or a 1:1 mixture of the two each at 130 mM, suggesting that the combination of a positively charged amino group and guanidinium group in the same molecule, as present in l-Arg, is key to effectively prevent protein destabilization at this interface (Figure S11b).

Analysis of samples spiked with mAb1-Alexa Fluor 647 by fluorescence microscopy showed that the addition of 130 mM l-Arg prevented the formation of a fluorescent protein rim (Figure 5c,d). These results indicate that l-Arg avoids antibody adsorption, with possible mechanisms including the shielding of attractive electrostatic interactions, the modulation of H-bonds, and the interaction with exposed hydrophobic groups of the antibodies.^67^

Given the observed effect of l-Arg, we analyzed the impact of 25 mM l-His, a commonly used buffer with a partially protonated imidazole side chain. We observed the same results obtained with sodium phosphate buffer (Figure S13). Moreover, an increase in the concentration of l-His to 130 mM decreased the strength of the viscoelastic layer strength to an extent similar to NaCl and l-Lys (Figure S11), indicating the absence of a protective effect of l-His in addition to electrostatics.

### 3.6 l-Arg Mitigates Protein Particle Formation at OA–Water Interfaces in High Antibody Concentration Formulations

**Figure 6:**
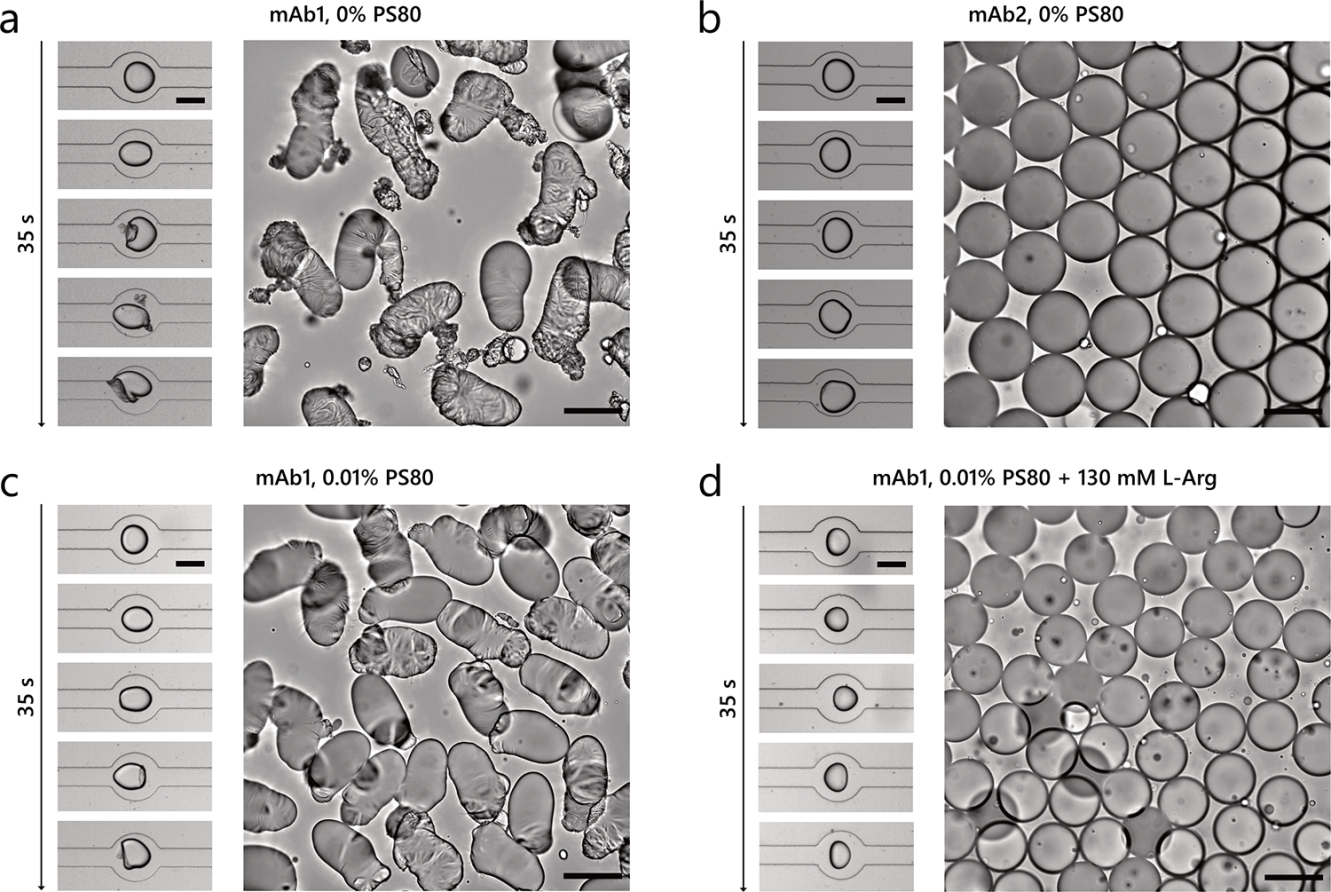
Brightfield microscopy images of OA droplets formed in the presence of 180 mg mL^–1^ (a) mAb1 and (b) mAb2 at pH 6.4 without surfactants. Panels (c) and (d) show OA droplets formed in the presence of 180 mg mL^–1^ mAb1 and (c) 0.01% PS80 and (d) 0.01% PS80 + 130 mM l-Arg. The left images show OA droplets at different expansion regions corresponding to residence times between 0.3 and 35 s, whereas the right images show the OA droplets after incubation for 1 h after collection from the chip. The scale bars are 100 μm.

The experiments discussed in the previous sections were performed at 30 mg mL^–1^. To analyze the effect of OA in conditions that are closer to formulations for subcutaneous administration,^68^ we measured the stability of the two antibodies at 180 mg mL^–1^ concentration at pH 6.4 in the absence of PS80. For mAb1, we observed progressive buckling of the viscoelastic protein layer and the shedding of particles into solution around OA droplets when traveling through the compression and expansion zones of the chip (Figure 6a and Movie S3). Moreover, even after 1 h of incubation, the OA droplets collected at the end of the chip did not relax back to a spherical shape and buckles remained at their interface, indicating the irreversible formation and collapse of the protein layer into solid-like particles. In contrast, for mAb2, OA droplets exhibited much less buckling on-chip and fully relaxed to a spherical shape off-chip (Figure 6b), showing no collapse of the adsorbed protein layer. The difference between the two antibodies at a high concentration is therefore consistent with the experiments performed at a lower concentration of 30 mg mL^–1^ (Figures 3b–d and S7).

The addition of 0.01% PS80 delayed but did not suppress the onset of buckling of the protein layer at the OA–water interface (Figure 6c). Buckling was still observed even at 0.05% PS80 at 180 mg mL^–1^ (Figure S14a) antibody concentration, in contrast to the results previously obtained at 30 mg mL^–1^ (Figure 4a). These observations indicate that at the higher protein concentration, the surfactant was only partially able to compete with the antibody for adsorption at the OA–water interface. This behavior was qualitatively predicted by the Langmuir model (eqs S4–S6 and Figure S10c,d), showing that a 6-fold increase in antibody concentration results in a rapid decay of surfactant coverage.

However, consistent with the same experiment with 30 mg mL^–1^ mAb1, the presence of 130 mM l-Arg, in both the absence and presence of 0.01% PS80 (Figures 6d and S14b), effectively prevented buckling and particle formation at 180 mg mL^–1^ mAb1 concentration.

## 4 Conclusions

Oleic acid (OA) is the primary free fatty acid resulting from the enzymatic hydrolysis of PS80 in pharmaceutical formulations. .

Here, we applied a droplet microfluidic device to investigate, for the first time, the interactions between therapeutic antibodies at concentrations up to 180 mg mL^–1^ and the water–oleic acid (OA) interface. In analogy to the silicone oil–water interface,^38^ we showed that antibody adsorption at the OA–water interface can lead to the formation of a viscoelastic protein layer on a time scale of seconds, which precedes particle formation upon mechanical perturbations such as syringe pumping.

We further demonstrated that two antibodies with similar bulk stability behavior but different zeta potentials and dipole moments exhibit different interfacial stability, underlining the role of protein surface physicochemical properties in modulating interactions with polar liquid interfaces.

We illustrated that the ability of intact PS80 to protect antibodies from the OA–water interface depends on the pH and concentration of antibodies, which compete for interfacial adsorption.

Importantly, the presence of l-Arg at a concentration typical of approved mAb products completely prevented the formation of the viscoelastic layer and detectable particles, even at high antibody concentrations. This work demonstrates the ability of l-Arg to protect therapeutic proteins at polar, negatively charged liquid interfaces such as OA, in addition to its other effects on antibody bulk properties, such as viscosity.

Overall, our findings indicate that OA droplets generated in antibody products undergoing enzymatic hydrolysis can lead to the formation of protein particles via an interface-mediated pathway. Depending on the physicochemical properties and concentration of the protein and buffer constituents, intact PS80 alone might not be sufficient to protect against protein aggregation. Additional mitigation strategies may include the optimization of protein physicochemical properties, pH, and the addition of l-Arg.

Droplet microfluidic platforms, potentially in combination with machine learning methods, can assist in this optimization and enable mechanistic insights into the stabilizing properties of various excipients under different formulation conditions, even at high protein concentrations, using low sample volumes in the microliter range.

## Supporting Information Available

Additional methodological details on droplet shape analysis (Figure S1); experimental data and fitting procedure of the determination of self-association parameters and melting temperatures (Figures S2–S4); degree of dissociation of OA calculated using the Henderson–Hasselbalch relationship (Figure S5); brightfield and fluorescence images of OA droplets before and after mechanical perturbation (Figure S6); effect of syringe pumping of OA droplets in mAb1 and mAb2 formulations (Figure S7); full data sets of OA droplet deformation vs time in the presence of PS80 (Figure S8); details on the modeling of competitive adsorption of mAb and surfactant (Table S1, eqs S1–S6, Figures S9, and S10); full data sets of OA droplet deformation vs time in the presence of NaCl, l-Arg, l-Lys and GdnHCl (Figures S11 and S12); OA droplet deformation vs time in the presence of 25 and 130 mM l-His (Figure S13); and brightfield microscopy images of OA droplets in mAb1 formulation at 180 mg mL^–1^ in the presence of 0.05% PS80 or 130 mM l-Arg (Figure S14) (PDF)
Exemplary movie of OA droplets in the microfluidic channel in the presence of 30 mg mL^–1^ mAb1 (Movie S1) (MP4)
Exemplary movie of OA droplets formed in the presence of 30 mg mL^–1^ mAb2, showing coalescence off-chip (Movie S2) (MP4)
Exemplary movie of OA droplets in the microfluidic channel in the presence of 180 mg mL^–1^ mAb1 (Movie S3) (MP4)
