# Supplementary material for: Mitigation Strategies against Antibody Aggregation Induced by Oleic Acid in Liquid Formulations
Source: Mol Pharm. 2024 Oct 24;21(11):5761–71. doi: 10.1021/acs.molpharmaceut.4c00754 (PMC11539069; doi:10.1021/acs.molpharmaceut.4c00754)
Supplement: Supplementary file 1 — mp4c00754_si_001.pdf [file mp4c00754_si_001.pdf]

Supporting Information

Mitigation Strategies Against Antibody Aggregation Induced by Oleic Acid-Water Interfaces

Dominik Zürcher[1], Klaus Wuchner[2], Paolo Arosio[1*]

[1] ETH Zürich, Department of Chemistry and Applied Biosciences
Institute for Chemical and Bioengineering
Vladimir-Prelog-Weg 1-5/10
8093 Zürich, Switzerland
Email Address: paolo.arosio@chem.ethz.ch

[2] Cilag GmbH International
a division of Johnson & Johnson
TDS-Biologics
Analytical Development
8200 Schaffhausen, Switzerland

[*] Correspondence to: Paolo Arosio, e-mail address: paolo.arosio@chem.ethz.ch

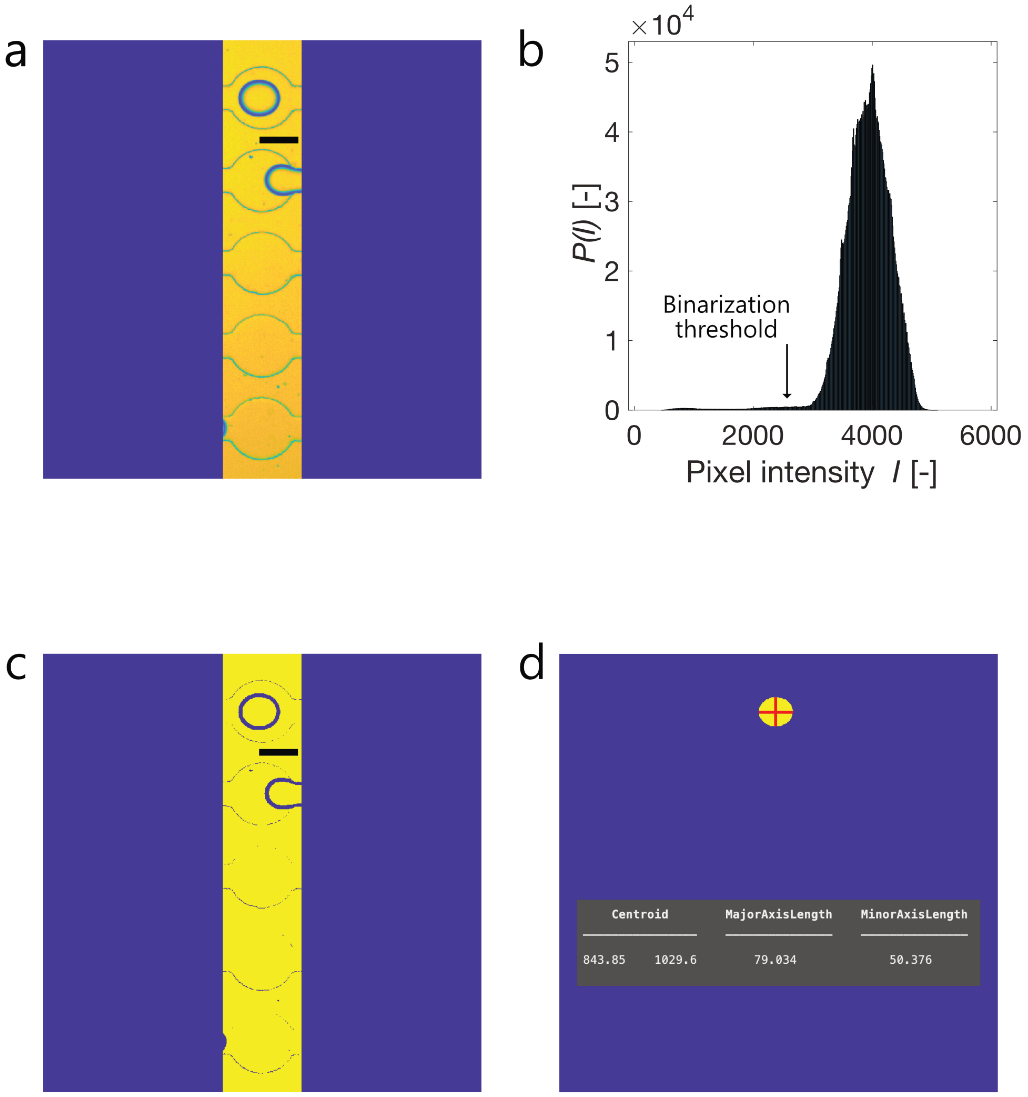

Figure S1: Illustration of the image analysis algorithm for the quantification of OA droplet shape from brightfield microscopy images acquired at different chip expansion regions at 4x magnification. a) Cropped, unmodified brightfield image showing expansion regions 10, 11, 34, 35 and 58 (from top to bottom). Scale bar is 100 µm. b) Intensity histogram of the image shown in a) and threshold (intensity = 2'500) to for image binarization. c) Binarized image, which allows to extract the droplet pixels from the background. Scale bar is 100 µm. d) Droplet shape extracted using a home-made Matlab code. The length of the major and minor axes (unit pixels) of the extracted droplet are shown in red and quantified in the insert, used to calculate the dimensionless droplet deformation $D = (w - h)/(w + h)$.

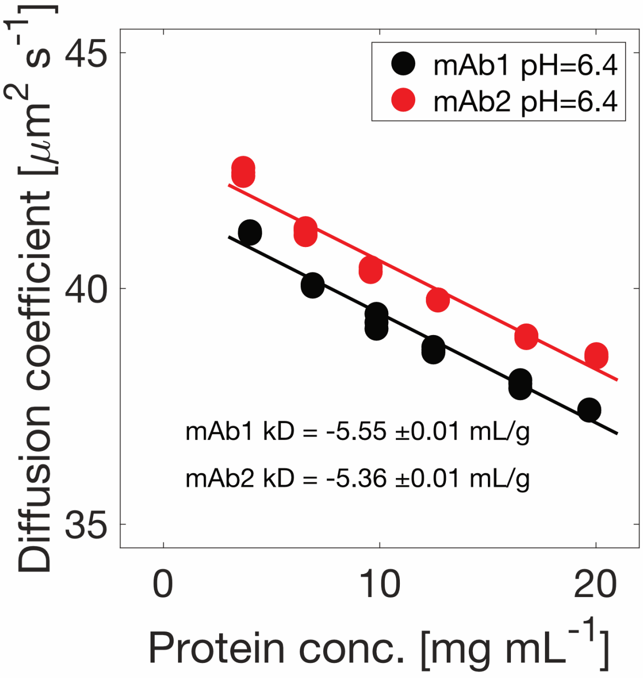

Figure S2: Diffusion coefficients of mAb1 and mAb2 in the concentration range of 3 to 20 mg mL^{-1} in the same buffer (6% trehalose, 44 mM sodium phosphate dibasic, 10 mM citric acid, pH 6.4) at 25°C as measured by dynamic light scattering. The solid lines represent linear fits according to the equation $D(c) = D_0 + k_D c$, where $D(c)$ represents the diffusion coefficient at concentration c, D_0 the diffusion coefficient at infinite dilution (intercept) and k_D the interaction coefficient (slope).

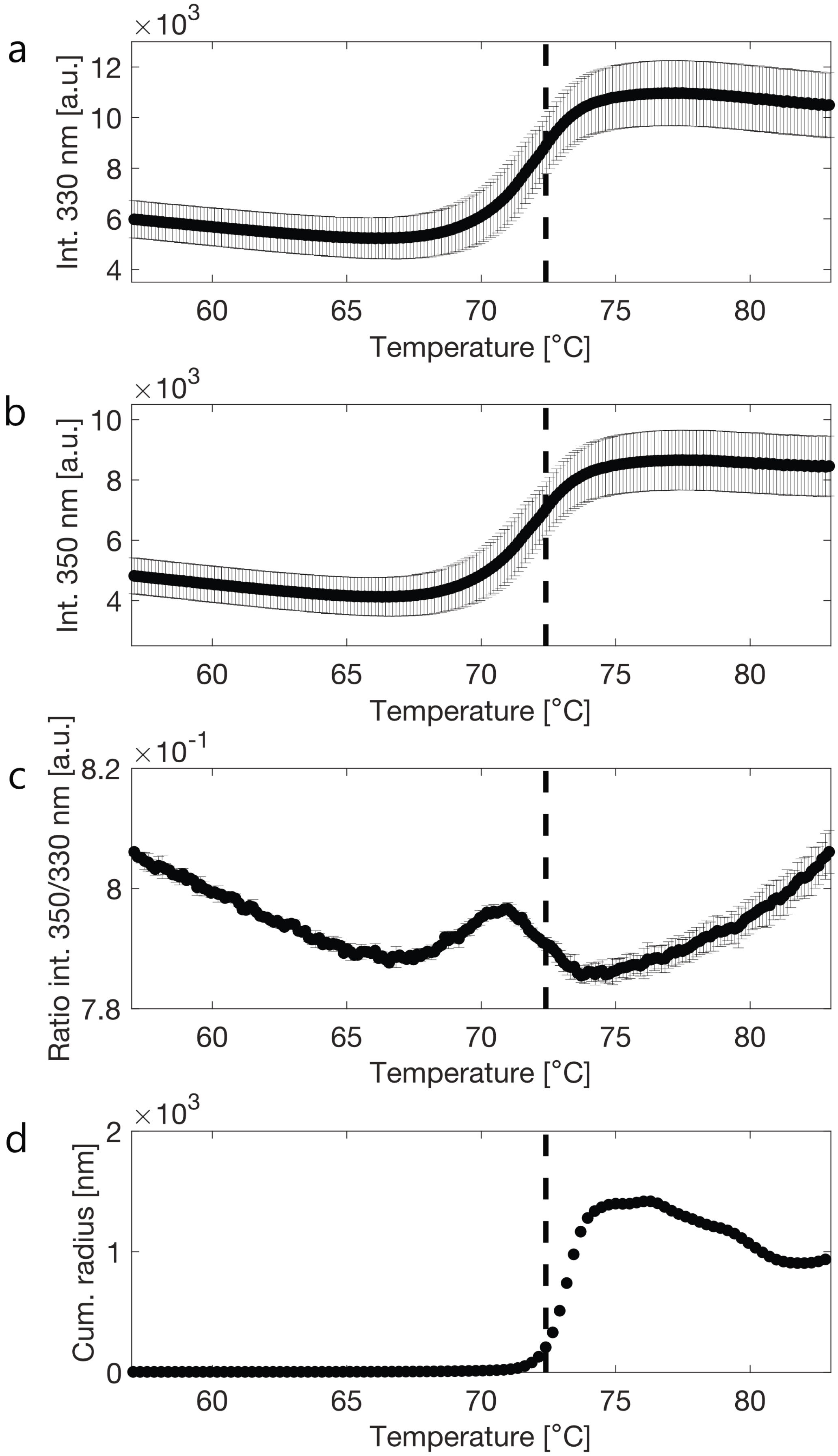

Figure S3: Thermal unfolding profiles of 6.9 mg mL^{-1} mAb1 at pH 6.4 showing fluorescence intensity at 330 nm (a), 350 nm (b), the ratio between fluorescence intensity at 350 and 330 nm (c), and the cumulant radius obtained by DLS (d). The dotted line represents the melting temperature of 72.4°C, obtained by a 2-state fitting of the raw data using the manufacturer's software.

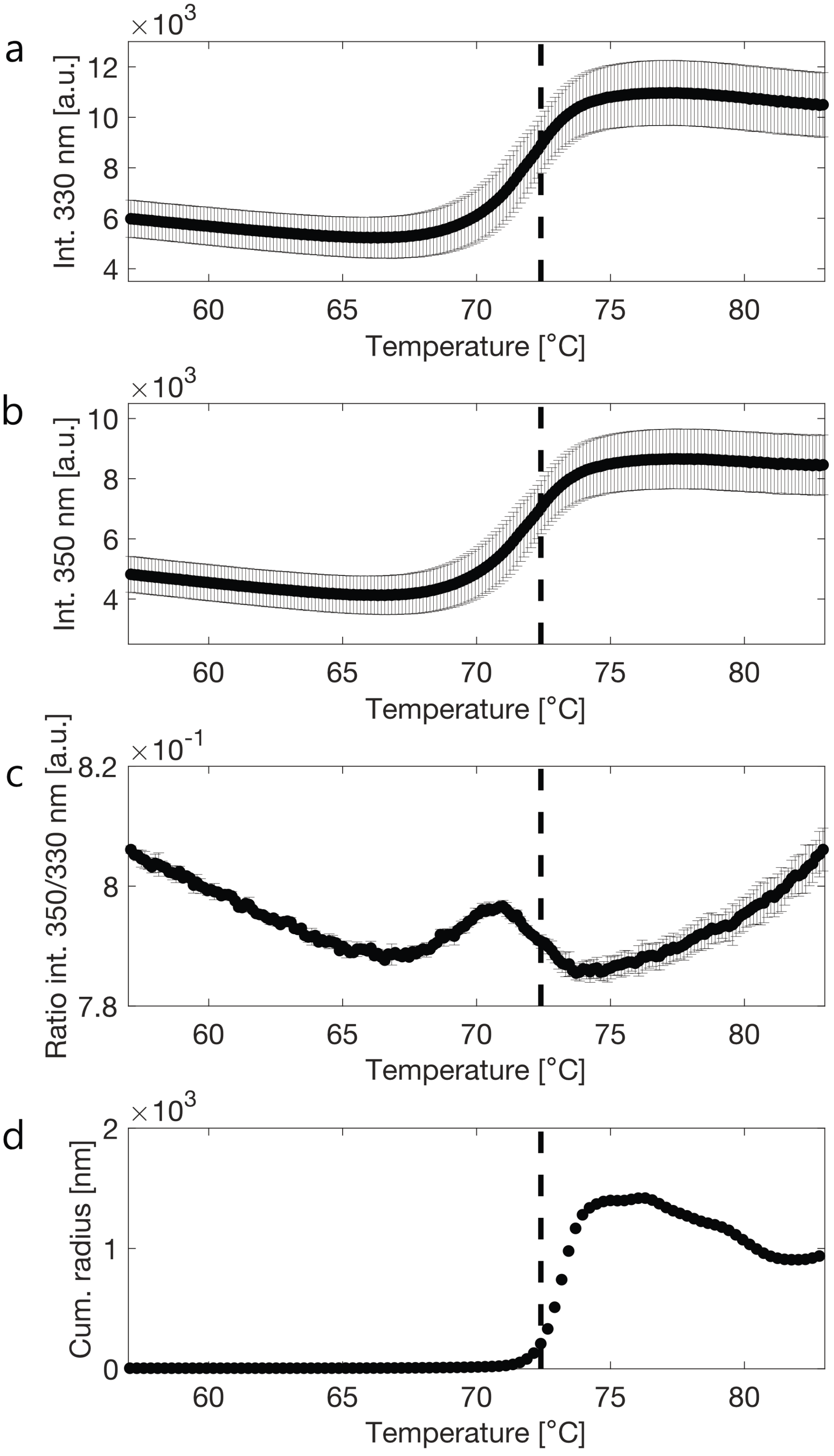

Figure S4: Thermal unfolding profiles of 6.6 mg mL^{-1} mAb2 at pH 6.4 showing fluorescence intensity at 330 nm (a), 350 nm (b), the ratio between fluorescence intensity at 350 and 330 nm (c), and the cumulant radius obtained by DLS (d). The dotted line represents the melting temperature of 70.0°C, obtained by a 2-state fitting of the raw data using the manufacturer's software.

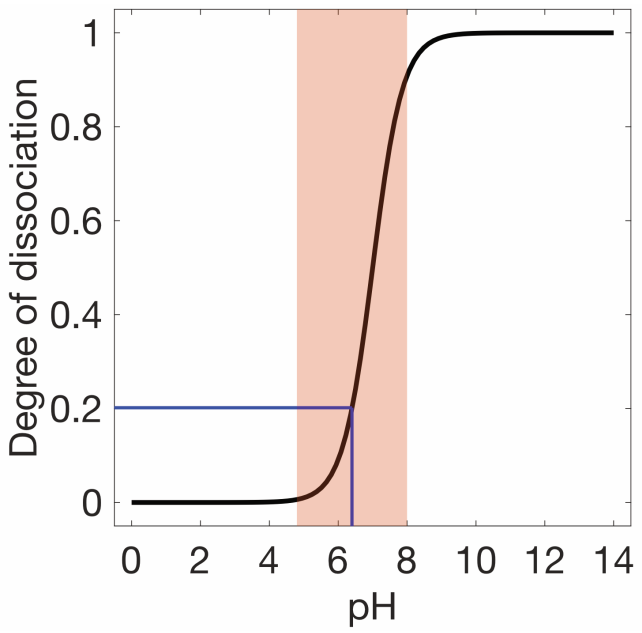

Figure S5: Degree of dissociation of oleic acid (OA) as function of solution pH according to the Henderson-Hasselbalch relationship, $1/(1+10^{\mathrm{pKa}-\mathrm{pH}})$, where the pKa of OA was assumed equal to 7.0.[1] The red area represents the typical range of pH values in commercial antibody formulations[2] while the blue line marks the degree of dissociation at a pH value of 6.4.

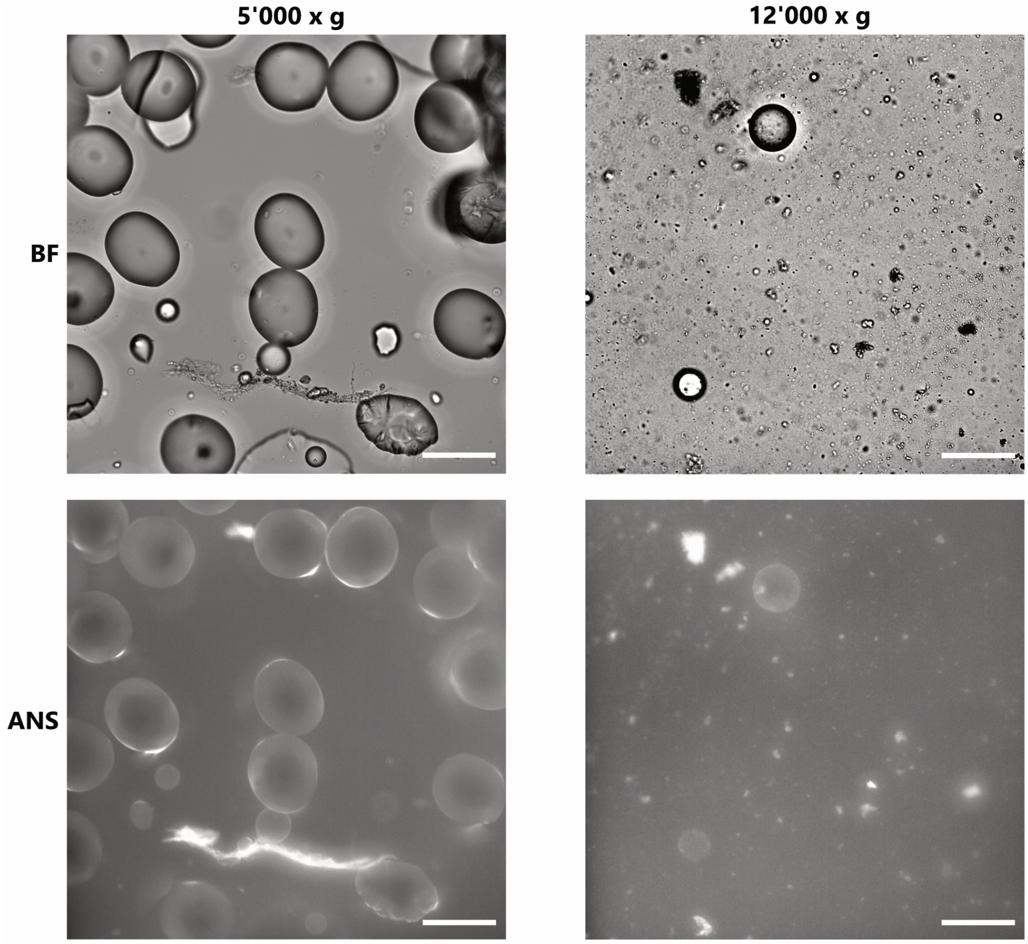

Figure S6: Brightfield (BF) and ANS-fluorescence (ANS) images of OA droplets formed in 30 mg mL^{-1} mAb1 formulation at pH 6.4 after centrifugation for 5 min at 5'000 x g and 12'000 x g. The extent of particle formation is drastically increased upon increased mechanical perturbation of the viscoelastic protein layer around OA droplets. Scale bars represent 100 µm.

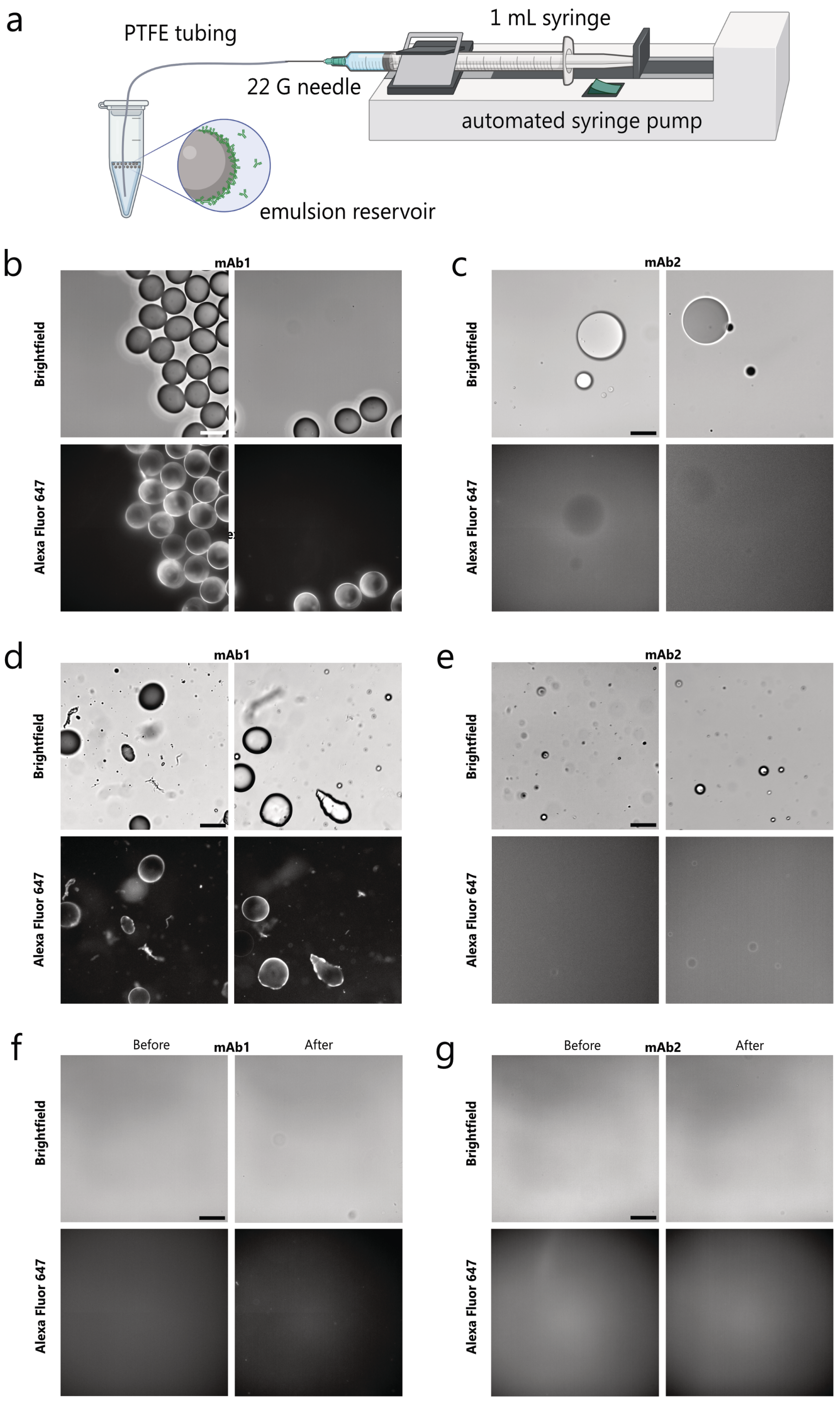

Figure S7: Effect of mechanical perturbation caused by syringe pumping of OA droplets formed in mAb1 and mAb2 formulation (30 mg mL^{-1}, pH 6.4) containing labeled mAb1-Alexa Fluor 647 or mAb2-Alexa Fluor 647, respectively, at a ratio of labeled to unlabeled protein of 1:700. a) Representation of the experimental setup. See also ref.[3] A polypropylene syringe (1 mL) (HSW Norm-Ject, Henke Sass Wolf) was filled with mAb1 or mAb2 formulation containing OA emulsion at a ratio of 10:1 (600 µL formulation without labeled protein + 60 µL emulsion formed on-chip in the presence of labeled protein). The syringe was connected to a reservoir (0.5 mL tube) via PTFE tubing and a 22 G needle. The syringe contents were expelled and aspirated 5 times at a rate of 0.5 mL min^{-1} using an automated syringe pump. Panels b) and c) show, respectively, representative brightfield and Alexa Fluor 647 fluorescence microscopy images of OA droplets before mechanical rupture generated in mAb1 (b) or mAb2 (c) formulation. OA droplets formed in mAb2 formulation showed coalescence to larger droplets (see also Movie S2) and a significantly reduced fluorescent rim compared to mAb1, indicating much lower enrichment of antibody at the interface. Panels d) and e) show representative images of OA droplets and continuous phase of mAb1 and mAb2 after 5 pumping cycles. f) and g) show control experiments of mAb1 and mAb2 formulation containing labeled antibody without the addition of OA droplets before and after 5 pumping cycles. The scale bar represents 100 µm.

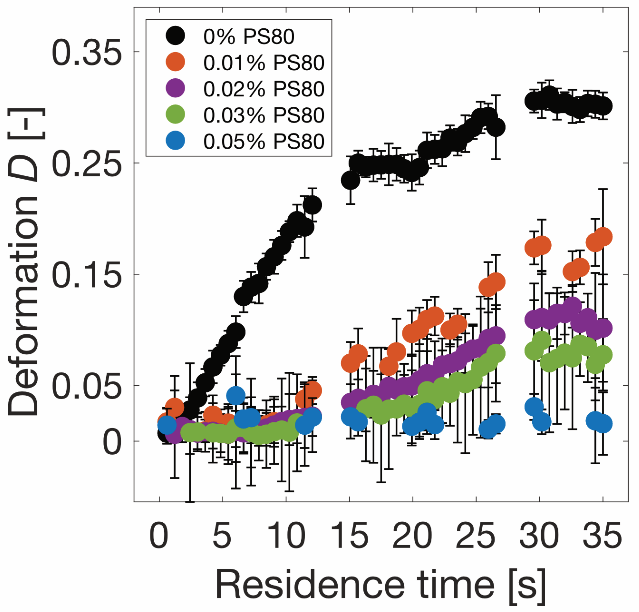

Figure S8: Deformation of OA droplets over time in the presence of mAb1 at 30 mg mL^{-1} and pH 6.4, supplemented with 0%, 0.01%, 0.02%, 0.03%, and 0.05% PS80.

Modelling of Competitive Protein and Surfactant Adsorption

In the limiting case where the arrival of protein molecules to the interface is controlled by diffusion, the transient surface concentration $\Gamma(t)$ can be related to protein bulk concentration C_P and the diffusion coefficient of the protein D_P by the following equation:[4]

$$\Gamma(t) = 2C_P \left(\frac{D_P t}{\pi}\right)^{1/2} \tag{S1}$$

Typical reported surface concentrations for monolayer saturation of antibodies adsorbed to the hydrophobic silicone oil-water interfaces span values between 0.5-5 mg m^{-2}.[5,6] Assuming similar surface concentrations at the OA interface and a typical diffusion coefficient of an antibody of $4 \cdot 10^{-11}$ m^2 s^{-1},[7] we find that the formation of a monolayer (5 mg m^{-2}) by diffusion to the interface requires approximately 0.5 ms at 30 mg mL^{-1} protein bulk concentration, which is well below the characteristic time of our experiment (35 s), indicating that interfacial adsorption and viscoelastic layer formation are not limited by diffusion under our experimental conditions.

In this case, as a first approximation, for protein and surfactant, the adsorption process is treated as a binding reaction between molecules in close proximity to the interface and a free binding site at the interface. The evolution of the surface coverage of each species i over time, $\theta_i(t) = \Gamma_i(t)/\Gamma_i^\infty$, can be expressed as[8]

$$\frac{\mathrm{d}\theta_i(t)}{\mathrm{d}t} = k_{i,\mathrm{ads}} C_{i,\mathrm{bulk}} \theta_i^*(t) - k_{i,\mathrm{des}} \theta_i(t) \tag{S2}$$

where $\Gamma_i(t)$, Γ_i^∞, $k_{i,\mathrm{ads}}$, $k_{i,\mathrm{des}}$ denote for each species the interfacial concentration, maximum interfacial concentration (max. number of binding sites), adsorption and desorption rate constant, respectively. $\theta_i^*(t)$ denotes the fractional coverage of free binding sites while $C_{i,\mathrm{bulk}}$ denotes the bulk concentration of each species. In the following, it is assumed that 1) Surfactant S and protein P compete for the same and equal number of binding sites, i.e., $\Gamma_S^\infty = \Gamma_P^\infty = \Gamma^\infty$ and $\theta_S^*(t) = \theta_P^*(t) = \theta^*(t)$; 2) The total number of binding sites, Γ^∞, is constant; 3) All binding sites are equivalent; 4) Mass transfer is fast such that the concentration of species in close proximity to the interface is equal to the bulk concentration. In contrast to the surfactant, the adsorption of protein to the interface is treated irreversibly, i.e., $k_{P,\mathrm{des}} = 0$.

Therefore, the material balance for the fractional coverage for adsorbed species reads:

$$\theta^*(t) + \theta_S(t) + \theta_P(t) = 1 \tag{S3}$$

and hence $\theta^*(t) = 1 - \theta_S(t) - \theta_P(t)$.

The system of ordinary differential equations (ODE) to model the competitive adsorption reads

$$\frac{\mathrm{d}\theta_S(t)}{\mathrm{d}t} = k_{S,\mathrm{ads}} C_{S,\mathrm{bulk}} \left(1 - \theta_S(t) - \theta_P(t)\right) - k_{S,\mathrm{des}} \theta_S(t) \tag{S4}$$

$$\frac{\mathrm{d}\theta_P(t)}{\mathrm{d}t} = k_{P,\mathrm{ads}} C_{P,\mathrm{bulk}} \left(1 - \theta_S(t) - \theta_P(t)\right) \tag{S5}$$

As an initial approximation to model droplet deformation as a function of adsorption, the rate of droplet deformation, $\frac{\mathrm{d}D}{\mathrm{d}t}$, is modeled to be linearly proportional to the rate of change of the fractional protein coverage $\frac{\mathrm{d}\theta_P(t)}{\mathrm{d}t}$ with proportionality constant K. We further introduce a critical surfactant fractional coverage $\theta_S^{\mathrm{crit.}}$, above which the droplet can fully relax to a sphere without requiring the fractional protein coverage to be zero. This corresponds to the situation where the adsorbed surfactant fully prevents the formation of a viscoelastic layer of co-adsorbed protein and hence deformation. The co-adsorption of antibodies and nonionic surfactant molecules at air and hydrophobic solid interfaces has been showed to occur using a variety of techniques including spectroscopic ellipsometry, neutron reflection and optical microscopy.[9,10]

$$\frac{\mathrm{d}D}{\mathrm{d}t} = \begin{cases} K\frac{\mathrm{d}\theta_P(t)}{\mathrm{d}t} & \text{if } \theta_S(t) < \theta_S^{\mathrm{crit.}} \\ 0 & \text{if } \theta_S(t) > \theta_S^{\mathrm{crit.}} \end{cases} \tag{S6}$$

Using a custom-made Matlab program, the model equations were solved and globally fitted to the experimental data obtained at $C_{S,\mathrm{bulk}}$ = 0%, 0.01% and 0.05% PS80 and $C_{P,\mathrm{bulk}}$ = 30 mg mL^{-1}, providing the fitted parameters $k_{S,\mathrm{ads}}$, $k_{S,\mathrm{des}}$, $k_{P,\mathrm{ads}}$, K and $\theta_S^{\mathrm{crit.}}$. Table S1 summarizes the range of initial values, best fits, and literature values (where available) for $k_{S,\mathrm{ads}}$, $k_{S,\mathrm{des}}$, $k_{P,\mathrm{ads}}$, K and $\theta_S^{\mathrm{crit.}}$.

Table S1: Parameters, literature values (where available), initial value range and best fits of the model described in equations (S4-S6) to the datasets.

**Parameter**	**Initial Value Range**	**Best Fit (**$R^2 = 0.97$**)**	**Literature Values**
$k_{S,\text{ads}}$ [L mol^{-1} s^{-1}]	$1 \cdot 10^3 - 20 \cdot 10^3$	$5.8 \cdot 10^3$	$6 \cdot 10^3$ (PS80, solid hexanedecane thiol interface)[11]<br>$18 \cdot 10^3$ (PEG-PFPE, liquid HFE 7500 oil interface)[8]
$k_{S,\text{des}}$ [s^{-1}]	$1 \cdot 10^{-3} - 1$	0.69	$6 \cdot 10^{-3}$ (PEG-PFPE, liquid HFE 7500 oil interface)[8]
$k_{P,\text{ads}}$ [L mol^{-1} s^{-1}]	$1 \cdot 10^2 - 20 \cdot 10^3$	340	*
K [-]	$0.1 - 1$	0.34	-
$\theta_S^{\text{crit.}}$ [-]	$0.1 - 1$	0.25	-

* literature values for the protein adsorption rate constant $k_{P,\text{ads}}$ to hydrophilic liquid interfaces vary strongly, between $3000 - 1.4 \cdot 10^6$ L mol^{-1} s^{-1}.[11]

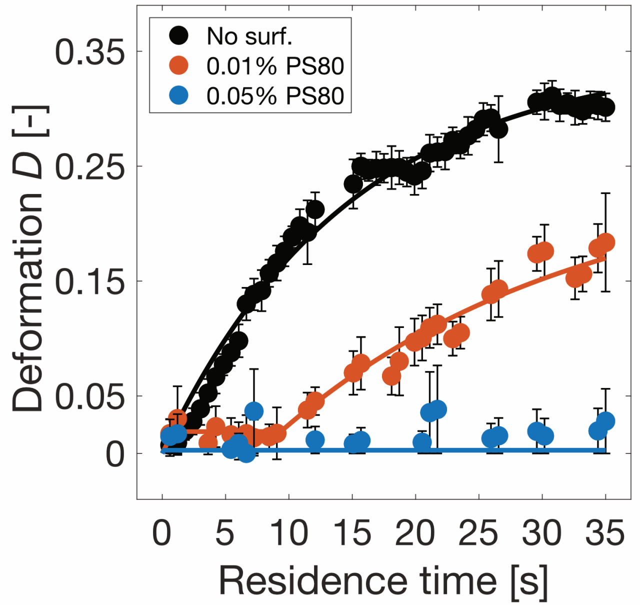

Figure S9: Best fits (R^2 = 0.97) obtained by global fitting of the model described in equations (S3)-(S6) to the experimental datasets obtained at pH 6.4 and mAb1 concentration of 30 mg mL^{-1} in the absence and presence of PS80 at 0.01% and 0.05%. Fitted parameters are shown in Table S1.

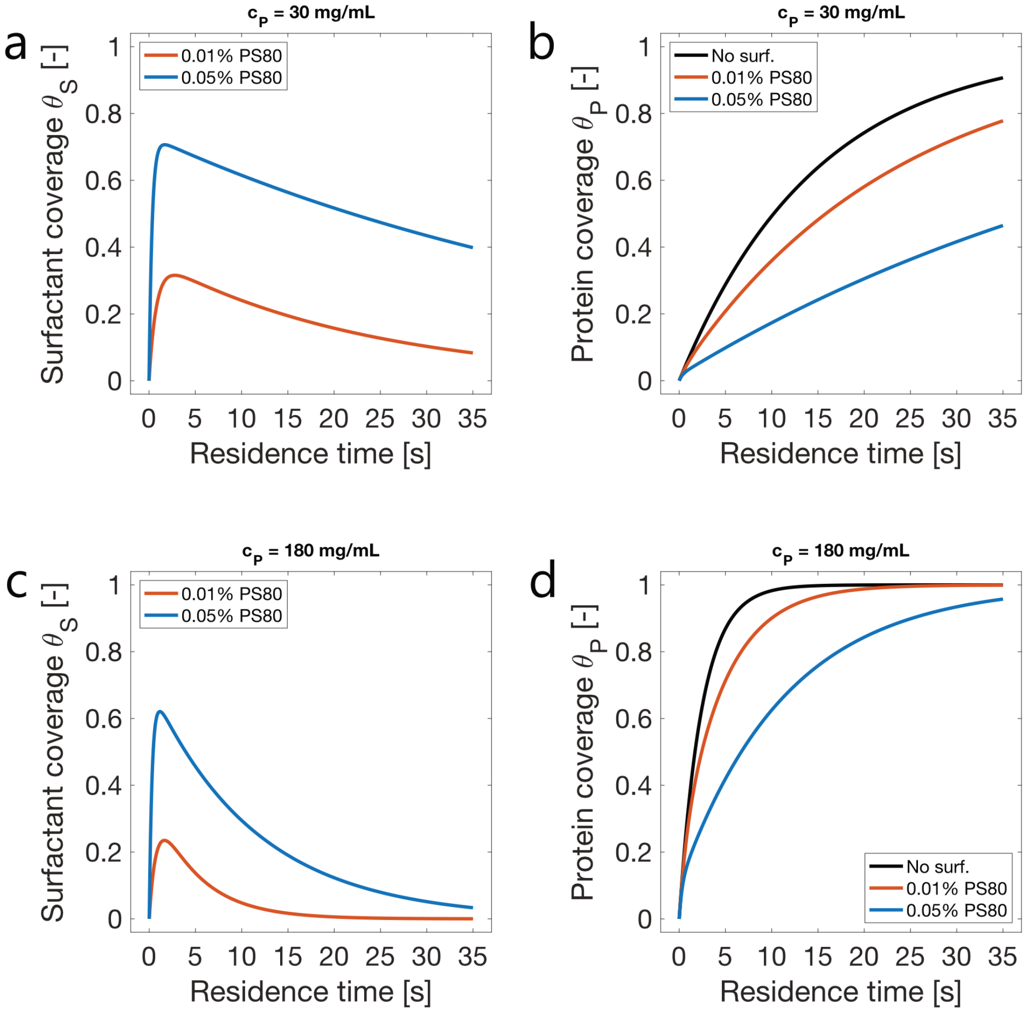

Figure S10: Fractional coverages of protein and PS80 at protein concentrations of 30 and 180 mg mL^{-1} and surfactant concentrations of 0, 0.01% and 0.05% according to equations (S4) and (S5) with the fitted values for $k_{S,\mathrm{ads}}$, $k_{S,\mathrm{des}}$ and $k_{P,\mathrm{ads}}$ given in Table S1.

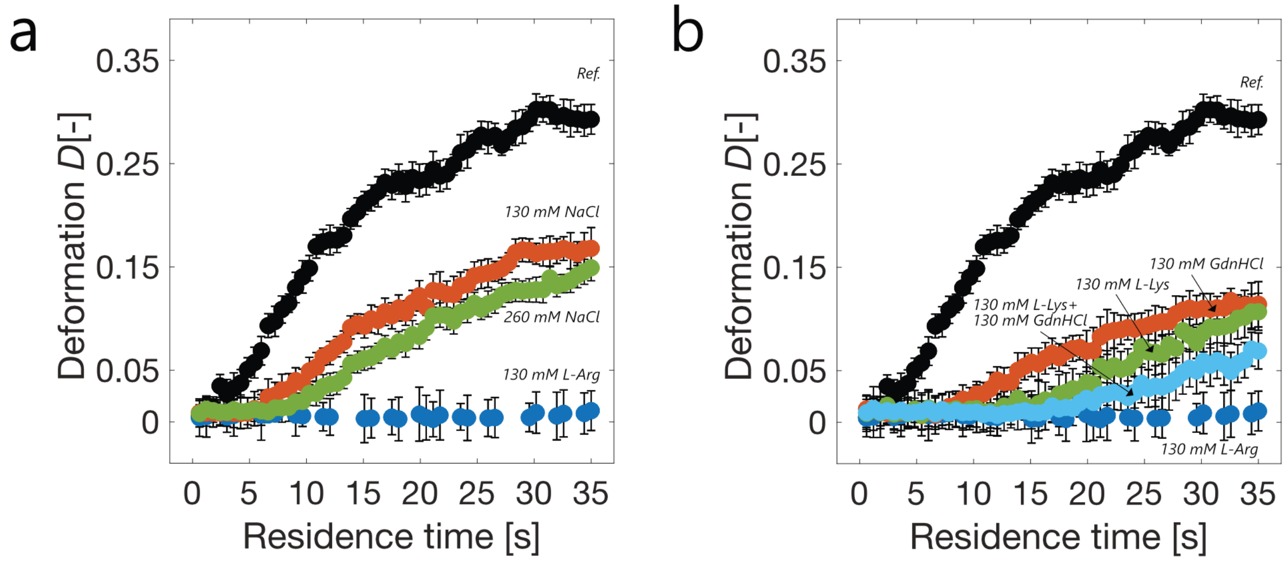

Figure S11: Deformation of OA droplets over time in the presence of 30 mg mL^{-1} mAb1 at pH 6.4, supplemented with a) 0 ("Ref."), 130 mM L-arginine (L-Arg), 130 or 260 mM sodium chloride (NaCl), and b) 0 ("Ref."), 130 mM L-arginine, 130 mM L-lysine (L-Lys), 130 mM guadinine hydrochloride (GdnHCl), or a combination of 130 mM L-lysine and 130 mM guadinine hydrochloride (L-Lys + GdnHCl).

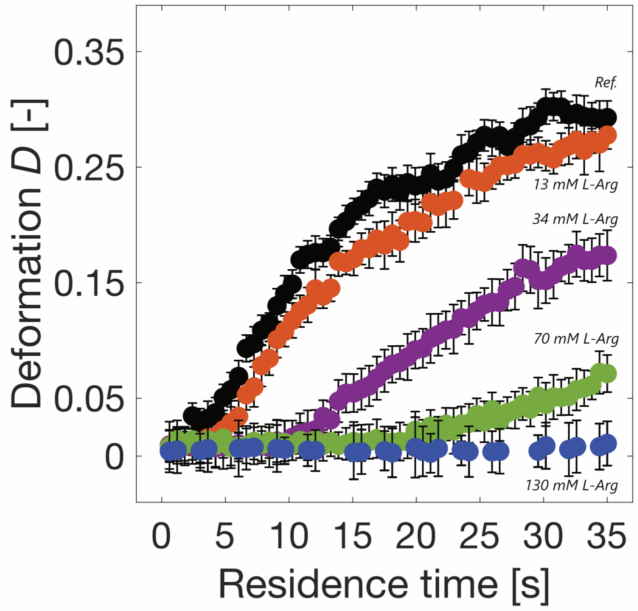

Figure S12: Deformation of OA droplets over time in the presence of 30 mg mL^{-1} mAb1 at pH 6.4, supplemented with 0 ("Ref."), 13, 34, 70 and 130 mM L-arginine.

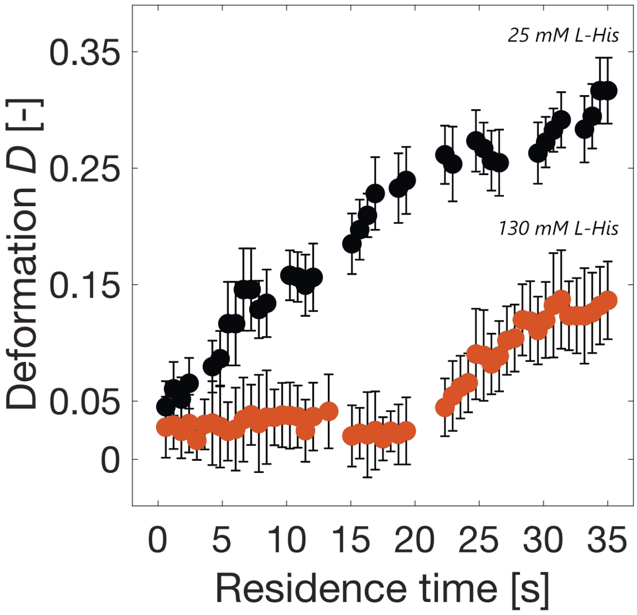

Figure S13: Deformation of OA droplets over time in the presence of 30 mg mL^{-1}mAb1 formulated in 6% D-trehalose, 25 or 130 mM L-histidine (L-His) at pH 6.4.

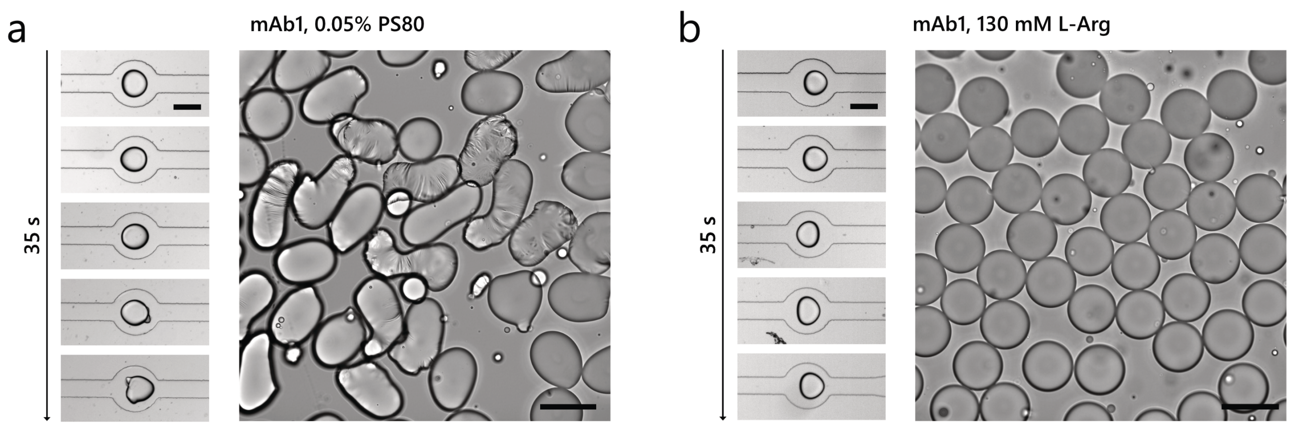

Figure S14: Brightfield microscopy images of OA droplets formed in 180 mg mL^{-1} mAb1 formulation at pH 6.4 in the presence of a) 0.05% PS80 and b) 130 mM L-Arg. The left images show OA droplets at different expansion regions corresponding to residence times between 0.3 and 35 s, whereas the right images show OA droplets after incubation for 1 hour after collection from the chip. Scale bars represent 100 µm.

References

[1] Doshi, N.; Demeule, B.; Yadav, S. Understanding particle formation: solubility of free fatty acids as polysorbate 20 degradation byproducts in therapeutic monoclonal antibody formulations. *Mol. Pharm.* **2015**, *12*, 3792–3804.

[2] Strickley, R. G.; Lambert, W. J. A review of Formulations of Commercially Available Antibodies. *J. Pharm. Sci.* **2021**, *110*, 2590–2608.e56.

[3] Grigolato, F.; Arosio, P. Synergistic effects of flow and interfaces on antibody aggregation. *Biotechnol. Bioeng.* **2020**, *117*, 417–428.

[4] Graham, D. E.; Phillips, M. C. Proteins at liquid interfaces. II. Adsorption isotherms. *J. Colloid Interface Sci.* **1979**, *70*, 415–426.

[5] Gerhardt, A.; McGraw, N. R.; Schwartz, D. K.; Bee, J. S.; Carpenter, J. F.; Randolph, T. W. Protein aggregation and particle formation in prefilled glass syringes. *J. Pharm. Sci.* **2014**, *103*, 1601–1612.

[6] Li, Y.; Pan, D.; Nashine, V.; Deshmukh, S.; Vig, B.; Chen, Z. Understanding protein-interface interactions of a fusion protein at silicone oil-water interface probed by sum frequency generation vibrational spectroscopy. *J. Pharm. Sci.* **2018**, *107*, 682–689.

[7] Beverung, C. J.; Radke, C. J.; Blanch, H. W. Protein adsorption at the oil/water interface: Characterization of adsorption kinetics by dynamic interfacial tension measurements. *Biophys. Chem.* **1999**, *81*, 59–80.

[8] Brosseau, Q.; Vrignon, J.; Baret, J. C. Microfluidic dynamic interfacial tensiometry (μDIT). *Soft Matter* **2014**, *10*, 3066–3076.

[9] Shen, K.; Hu, X.; Li, Z.; Liao, M.; Zhuang, Z.; Ruane, S.; Wang, Z.; Li, P.; Micciulla, S.; Kasinathan, N.; Kalonia, C.; Lu, J. R. Competitive Adsorption of a Monoclonal Antibody and Nonionic Surfactant at the PDMS/Water Interface. *Mol. Pharm.* **2023**, *20*, 2502–2512.

[10] Kannan, A.; Shieh, I. C.; Fuller, G. G. Linking aggregation and interfacial properties in monoclonal antibody-surfactant formulations. *J. Colloid Interface Sci.* **2019**, *550*, 128–138.

[11] Lefebvre, G.; Bruckert, F.; Filipe, V.; Huille, S.; Weidenhaupt, M. Adsorption rate constants of therapeutic proteins and surfactants for material surfaces. *Colloids Surfaces B Biointerfaces* **2021**, *203*, 111722.
